# A Chemical Reaction Network Drives Complex Population Dynamics in Oscillating Self-Reproducing Vesicles

**DOI:** 10.1021/jacs.4c00860

**Published:** 2024-06-25

**Authors:** Zhiheng Zhang, Michael G. Howlett, Emma Silvester, Philipp Kukura, Stephen P. Fletcher

**Affiliations:** † Chemistry Research Laboratory, Department of Chemistry, University of Oxford, 12 Mansfield Road, Oxford OX1 3TA, U.K.; ‡ The Kavli Institute for NanoScience Discovery, Dorothy Crowfoot Hodgkin Building, Oxford OX1 3QU, U.K.; § Department of Biochemistry, University of Oxford, Oxford OX1 3QU, U.K.; ∥ Physical and Theoretical Chemistry Laboratory, Department of Chemistry, University of Oxford, South Parks Road, Oxford OX1 3QZ, U.K.

## Abstract

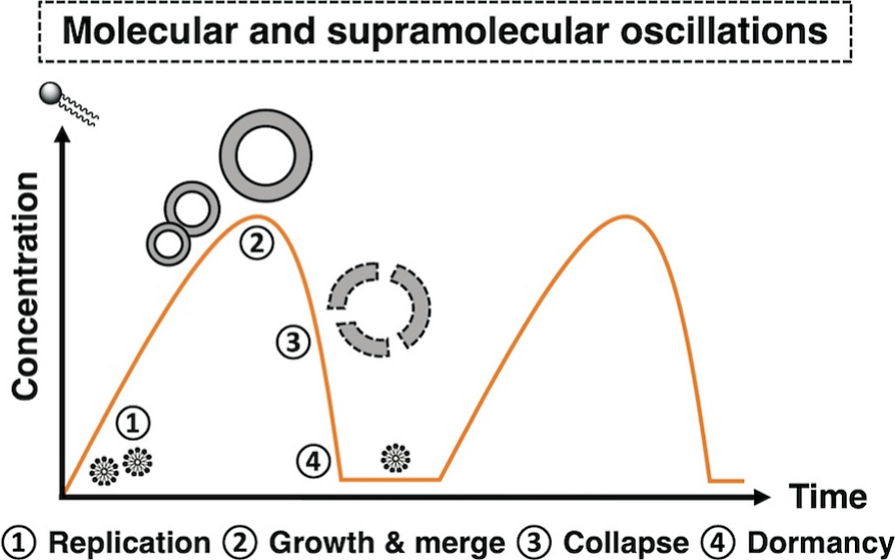

We report chemically fueled oscillations of vesicles. The population cycling of vesicles is driven by their self-reproduction and collapse within a biphasic reaction network involving the interplay of molecular and supramolecular events. We studied the oscillations on the molecular and supramolecular scales and tracked vesicle populations in time by interferometric scattering microscopy and dynamic light scattering. Complex supramolecular events were observed during oscillations—including vesicle reproduction, growth, and decomposition—and differences in the number, size, and mass of aggregates can often be observed within and between pulses. This system’s dynamic behavior is reminiscent of a reproductive cycle in living cells.

## Introduction

The complexity and intricacy of living systems inspire scientists to understand how molecular structures may achieve life-like dynamics and functions.^1,2^ Despite numerous hypotheses regarding the origins of life,^3−7^ it is still unclear how simple molecules can give rise to the dynamics observed in living systems which can control functions across the molecular, microscopic, and macroscopic scales.

Among the diverse behaviors characterized in living systems, those that are periodic in time are particularly fascinating. Oscillations are seen in events as disparate as glycolysis,^8^ expression of tumor suppressor protein p53,^9^ circadian rhythms,^10^ and the reproductive cycle of cells.^11^

Living cells have the ability to perform complex, time-controlled functions to sustain themselves, synchronize, and respond to external stimuli. Many models have been developed in order to simulate and better understand the operations of living cells.^12,13^ However, most artificial cells are either static structures or need repeated or continuous stimulation to maintain their structures and control functions.^14−17^ In living cells, various feedback mechanisms enable autonomous cellular processes, and many internal biochemical reactions exhibit periodic behavior.^18^

Designing oscillating chemical reactions may be relevant to understanding periodicity in biology. The mathematical and physical foundations of nonequilibrium systems have been extensively discussed,^19,20^ but it is not at all well understood how to incorporate the specific design elements required to observe nonequilibrium systems into chemical reaction networks so that novel systems may oscillate and show complex dynamic behavior.

Multiple approaches have been used to hold chemical systems out-of-equilibrium, including the addition of chemical fuels,^21^ light,^22^ and electrical energy.^23^ Our group has also developed several out-of-equilibrium supramolecular systems,^24^ including chemically fueled self-reproducing micelles^25^ and vesicles,^26^ and we have observed competition and selection in systems containing mixtures of protocells^27^ and characterized lipid populations that evolve in time.^28^

Several oscillating chemical reactions have been discovered over the last 200 years.^29^ While classical oscillators feature electrochemical and inorganic components,^30−33^ more contemporary examples are based on chemo-mechanical systems,^34^ supramolecular polymers,^35^ and there are several systems where continuous-flow stirred tank reactors (CSTRs) are used to drive oscillations.^36−40^ CSTRs constantly replenish starting materials and remove products while maintaining a fixed volume and are the major experimental tool for developing out-of-equilibrium systems and rationally designed oscillators.^41,42^

**Figure 1:**
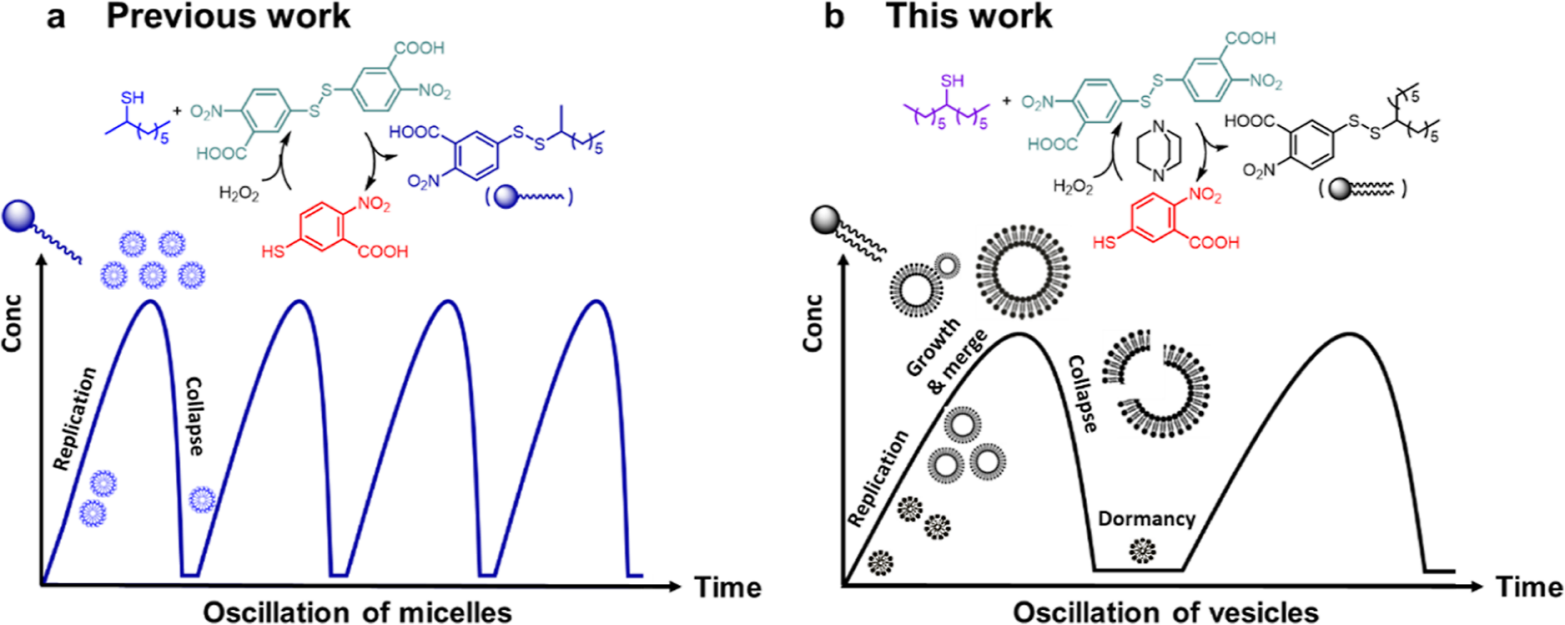
(a) Previous work on oscillating self-replicating micelles. (b) This work: oscillating self-replicating vesicles.

In 2022, our group reported oscillating micelles (Figure 1a).^43^ This system depends strongly upon nonlinear feedback mechanisms between micelles and their phase-separated precursors.^44^ Rather than mechanical flow, our system uses a chemically fueled reaction network to regenerate reactive starting materials from a waste product of the reaction, in a manner that is similar to the metabolic cycles seen in biology.

Vesicles are widely regarded as basic compartments that may be relevant to the origins of life and are likely closer to living systems than micelle-based systems.^45,46^ Light-driven oscillations in a vesicle-forming system have been previously reported by Pérez-Mercader and co-workers.^47,48^ In that system, light-induced radical polymerization led to the formation of giant polymer vesicles, which showed “Phoenix behavior” (growth, collapse, and regrowth) and phototaxis.

We previously reported that self-reproducing metastable vesicles could be kept in a steady state.^26^ Here, we set out to develop a system of vesicles that could oscillate over time (Figure 1b).

## Results and Discussion

### Molecular Oscillations

**Figure 2:**
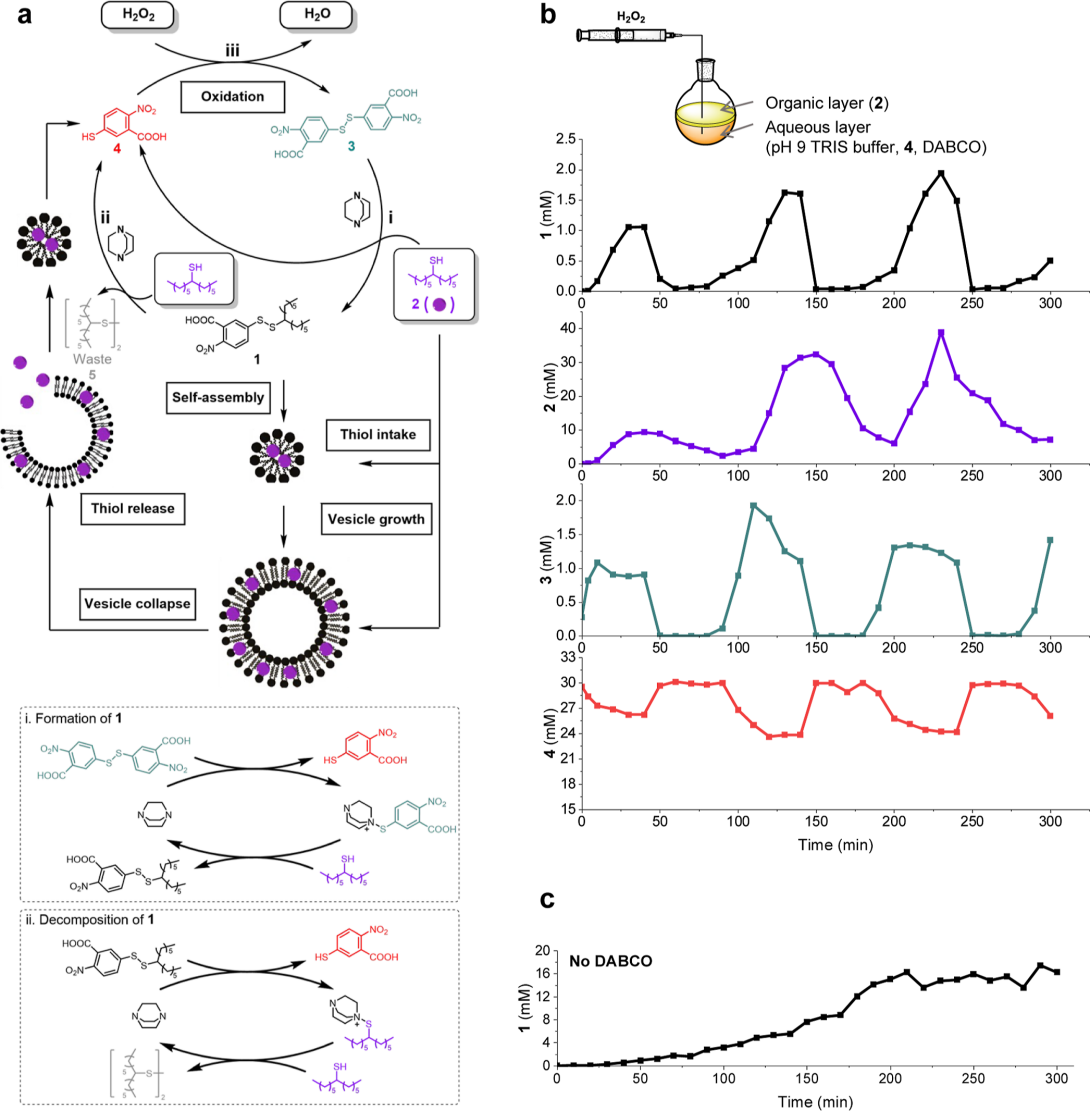
(a) Chemical reactions and supramolecular events in the cycle of oscillating vesicles. Surfactant **1** is formed in the reaction of phase-separated thiol **2** and disulfide **3**; waste product **4** is also formed [step (i)]. Surfactant **1** aggregates to form supramolecular species; the formation of **1** is autocatalytic because aggregates of **1** allow **2** and **3** to mix. Disulfide **3** is the limiting reagent in the system and in the absence of **3**, surfactant **1** and disulfide **2** react [step (ii)] to give waste products **4** and **5**. The decomposition of molecular **1** also decomposes aggregates of **1** and this step is also nonlinear. The constant addition of hydrogen peroxide converts waste **4** back into electrophilic disulfide **3** [step (iii)]. As shown in boxes (bottom) for steps (i) and (ii), DABCO mediates both formation (i) and decomposition (ii) of **1**. (b) Top: Cartoon picture of the oscillating biphasic reaction, where a mixture of **2** and aqueous **4** is stirred while H_2_O_2_ is added at a constant rate. Bottom: Aqueous phase concentrations of **1**, **2**, **3**, and **4** as determined by removing aliquots of the reaction, quenching with 0.06 M aqueous maleimide and measurement by ultraperformance liquid chromatography (UPLC). The molecular concentration of surfactant **1** oscillates. We assume some **1** is present in the organic phase, which is undetected when the aqueous layer is sampled and analyzed. The concentration of **2** is largely in phase with **1**, but it lags somewhat, consistent with **1** carrying **2** into the aqueous phase. The concentrations of **3** and **4** are out of phase. DABCO adducts [Figure 2a, steps (i) and (ii)] are expected to be undetected and lower the measured concentration of **4**. The concentration of **4** returns to ∼30 mM after every pulse and so appears to be completely regenerated. (c) In the absence of DABCO, **1** forms slowly and the system did not oscillate on the time scale examined. Lines are drawn to guide the eyes.

The key to observing oscillations in the self-replicating vesicles shown here is a metabolic cycle (Figure 2a) that replenishes starting material from a waste product of the reaction. The metabolic cycle features autocatalytic steps for both formation and decomposition of **1**.

Surfactant **1** is formed when **2** reacts at the interface with **3**. Molecular **1** aggregates to form vesicles in an aqueous buffer at pH 9.0. The formation of **1** is autocatalytic because aggregates of **1** transport **2** from the organic layer to locations where it can more readily react with **3** to produce more **1**. This physical autocatalytic mechanism operates on the principle that the products of the reaction increase the reaction rates between phase-separated precursors.^24,26,44^

The system is biphasic, with thiol **2** initially comprising the organic layer, and **4** and DABCO initially dissolved in aqueous buffer. The continuous addition of H_2_O_2_ via a syringe pump forms **3** from **4** and keeps the system out of equilibrium.

Electrophilic **3** is the limiting reagent in the autocatalytic formation of **1**. When **3** is depleted, nucleophilic **2** (solubilized by aggregates of **1**) reacts with electrophilic **1**—and the decomposition of **1** causes the vesicles to collapse and forms waste products **4** and **5** in a second autocatalytic process. While the asymmetry of the peak shapes in the kinetics of **1** shows that vesicle collapse is a highly nonlinear process, the autocatalytic mechanism at play here is not necessarily obvious. At this stage, we believe that the reason the decomposition of **1** is autocatalytic is because the partial collapse of the supramolecular structure releases thiol **2** into the aqueous phase, which is more active than phase-separated thiol and reacts readily with **1** to further promote vesicle destruction.^43^

The surfactant formation and destruction phases are both autocatalytic, and the molecular concentrations of reactants **1**–**4** oscillate (Figure 2b). As well as constantly supplying peroxide and stirring, the presence of a nucleophilic catalyst (DABCO) is also required to see oscillations (at least on the time scales examined), as oscillations require a balance of reaction rates in the formation and decomposition of the reaction components, and while this may be obtainable by other means (for example, stirring speed), we found the use of a nucleophilic catalysis convenient and possibly relevant to how relative reaction rates may be fine-tuned in biological systems. While DABCO likely catalyzes both formation [Figure 2a(i)] and decomposition [Figure 2a(ii)] of **1**, we propose that it plays a key role in the oscillations by catalyzing the decomposition of **1**, as the formation of **1** is still observed in the absence of DABCO (Figure 2c).

### Supramolecular Oscillations

We used interferometric scattering microscopy (iSCAT) to detect supramolecular species in real-time during oscillations. iSCAT can measure the change in refractive index caused by particles landing or unbinding on a surface, with the change in contrast being proportional to particle mass.^49^ This powerful method has been used to analyze micelles and vesicles in situ.^26,43,50^ Dynamic light scattering (DLS)^51^ was used to measure the average size of the vesicles during oscillations, and cryo-TEM was used to observe the vesicles at equilibrium.

In a typical oscillation experiment, the average particle count and contrast were measured by iSCAT every 20 min (see Supporting Information page 4). The contrast values, whether positive or negative, signify unbinding and binding events of particles, with binding predominating in this experiment. The approximate particle mass is determined through the calibration curve in Figure S2.

**Figure 3:**
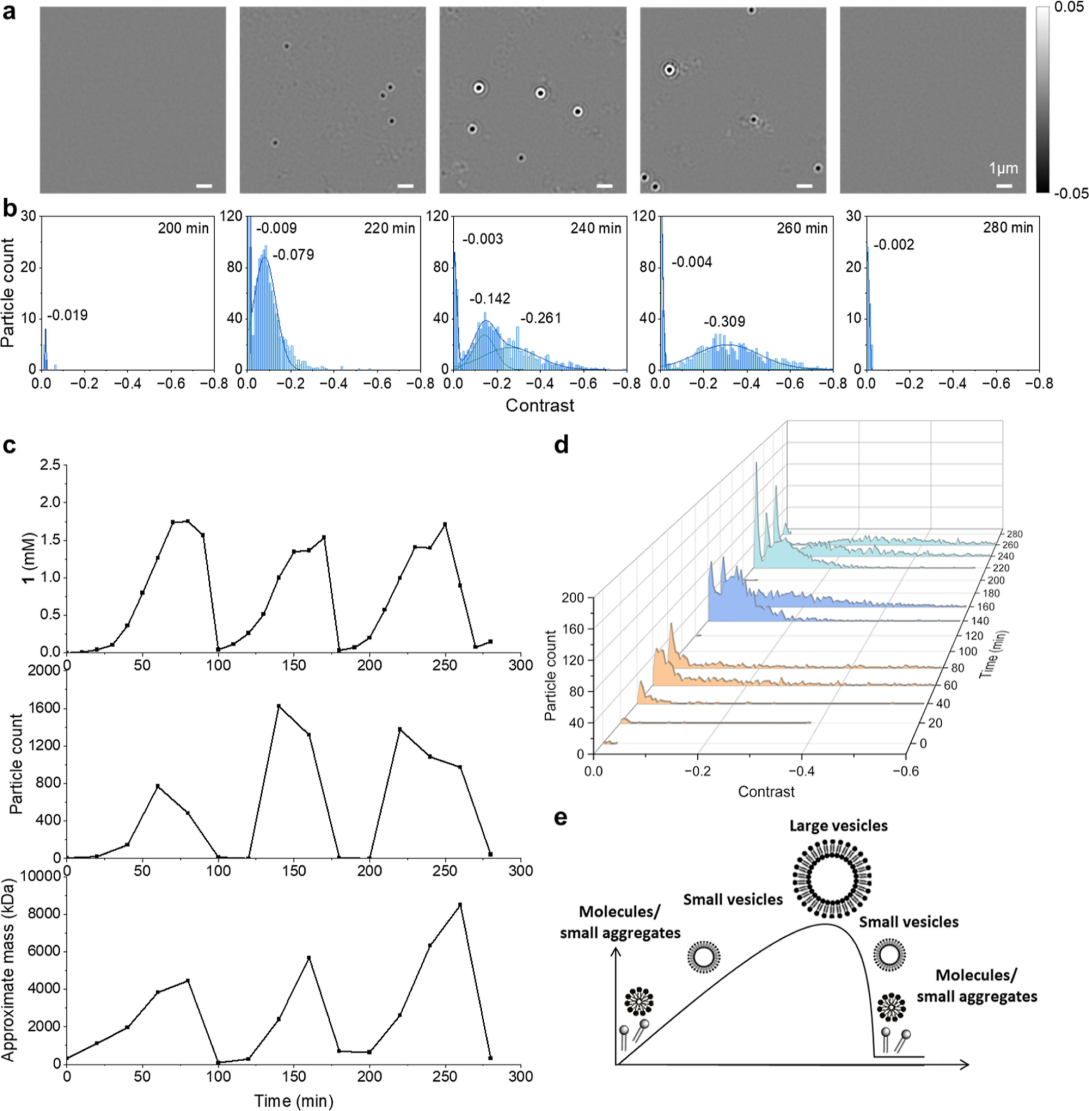
(a) iSCAT photos corresponding to the timepoint shown in panel (b) with contrast threshold of ±0.05 showing that micelles or small vesicles are first formed, then large vesicles are observed before the supramolecular aggregates decay. The scale bar is 1 μm. (b) Fitted Gaussian curve of contrast distribution within one oscillation pulse where the changes in distribution suggest vesicles are growing. (c) The change in the concentration of 1, particle count, and the approximate average mass of particles over time during an oscillation experiment. Particle data were obtained from iSCAT movies. (d) Waterfall plot of contrast distribution over time, showing the change in particle count and contrast distribution over time. The data from 3 peaks are labeled by different colors. (e) Cartoon diagram of the supramolecular species observed during a single pulse of an oscillation. Lines are drawn to guide the eyes.

The iSCAT images clearly suggest that vesicles form, grow, and decay within one pulse of an oscillation (Figure 3a shows one pulse from 200 to 280 min). Vesicles appear as dark circles with a white boundary, with larger and brighter particles representing higher mass aggregates.

The change in contrast distribution over time is shown in Figure 3b (one pulse) and Figure 3d (overall), where all distributions are calculated based on a 60 s video at each time point. Low contrast values such as 0.0 to (−)0.1 indicate smaller assemblies such as micelles whereas greater values such as >(−)0.2 support the presence of larger aggregates such as vesicles.^26,50^ Initially in the reaction, mostly very low contrast values <(−)0.05 are seen, alongside a growing broad peak with medium contrast (∼(−)0.1). Over time, the contrast distribution broadens and shifts to higher contrast (>(−)0.25) during each oscillation pulse and in comparison to subsequent pulses. This supports the formation of larger and heterogeneous vesicle-scale aggregates during each pulse (in line with increasing **1**), similar to that previously observed,^26^ and is compared with a symmetric distribution and lower contrast seen for the analogous, smaller and more homogeneous micellar systems.^50^ Interestingly, the overall increase in average contrast shows that in this particular experiment (vide infra), larger aggregates are preferentially formed as the oscillation progresses, despite the apparent decay of aggregates between pulses.

In Figure 3c, the counts of all landing particles within 60 s are recorded, while the approximate average mass is calculated based on the average particle contrast. Simultaneously monitoring oscillations with iSCAT and UPLC revealed a correlation between the count and mass of the particles and the solution concentration of **1**. However, initially, the growth of count and mass lags slightly because the self-assembly process requires accumulation of sufficient building blocks **1** to reach the critical assembly concentration (Figure S3). A cartoon interpretation of the supramolecular dynamics—where the aggregates change during the course of a single pulse—is shown in Figure 3e.

**Figure 4:**
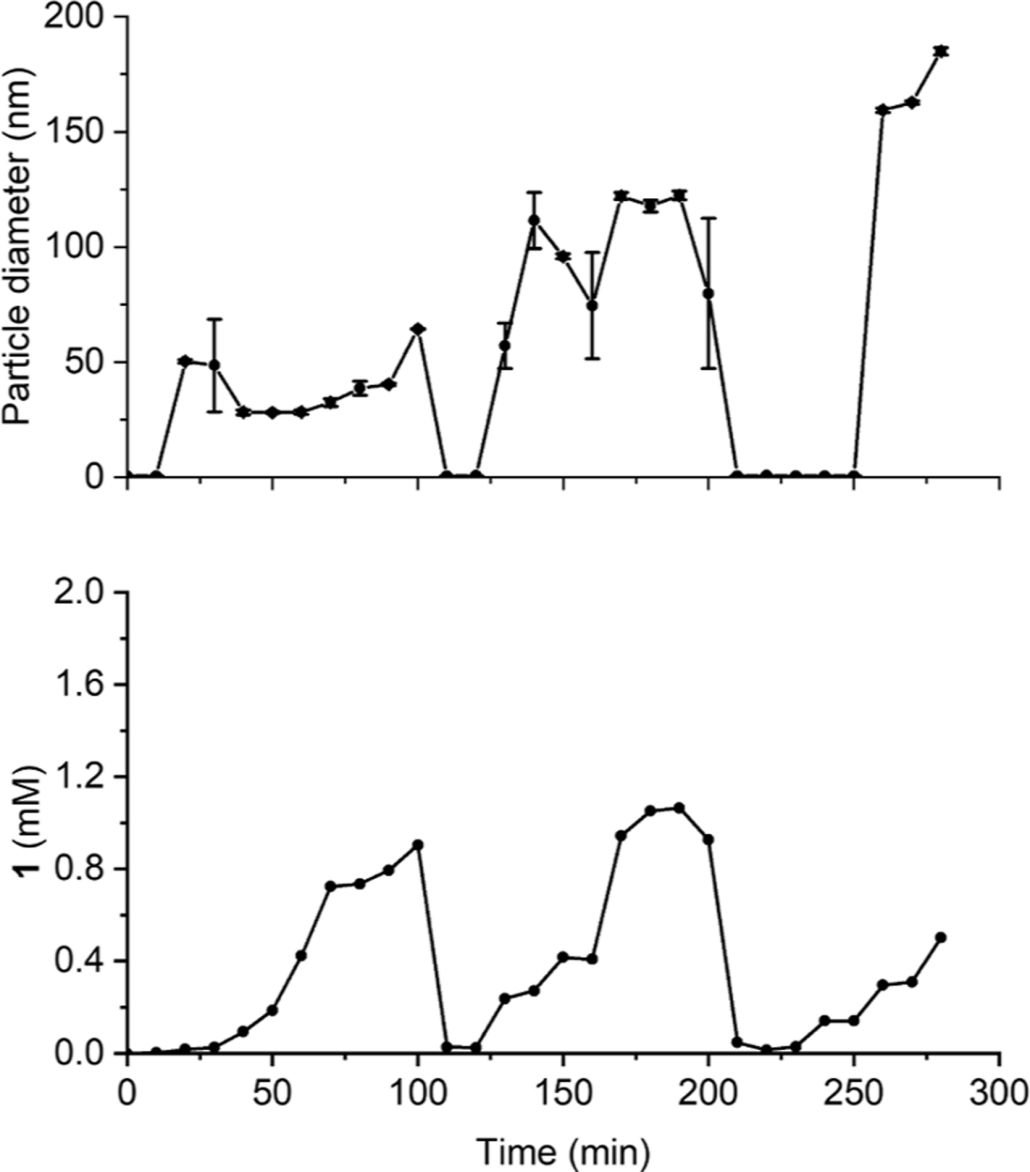
Number mean diameter of vesicles in time as measured by DLS. Lines are drawn to guide the eyes.

**Figure 5:**
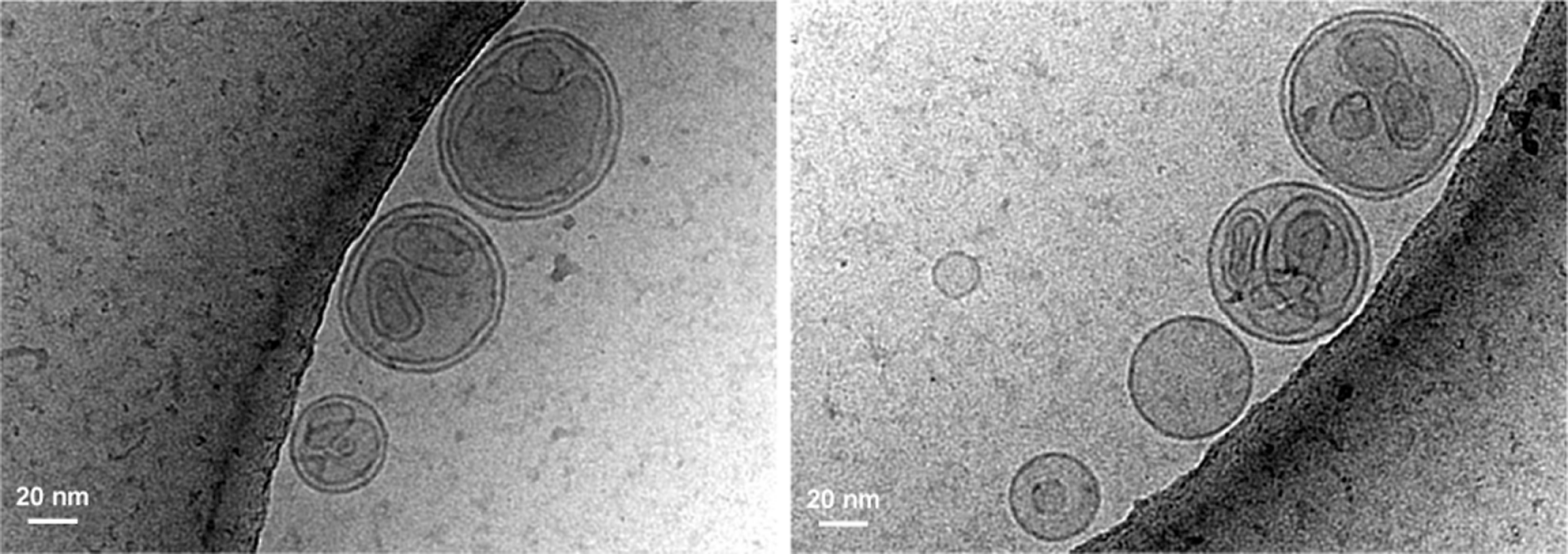
Cryo-TEM images of the vesicles of **1**. Images were recorded for 2.5 mM samples of **1** in a TRIS buffer (0.5 M, pH 9.00). The images show vesicles, including oligolamellar and multivesicular vesicles with a wide distribution of sizes.

While iSCAT provides a measure of the amount of material in a nanoscopic object, extracting the size is difficult for subdiffraction objects in the absence of other measures, such as diffusion. To track changes in the vesicle size, we monitored a reaction using DLS. Like with iSCAT experiments discussed below, reaction aliquots were taken every 10 min and then diluted to measure the particle size distribution over time. Figure 4 shows how the mean particle diameter (top) and the molecular concentration of **1** (bottom) fluctuate. Both DLS and iSCAT observations show the growth and decay of the vesicles. However, DLS measures hydrodynamic diameter, while iSCAT measures the amount of material, which is not necessarily trivially correlated. Cryo-TEM images (see Figure 5 and Supporting Information Figure S9) provide insights into how vesicles can increase in mass, but not size, as they show the formation of vesicles within vesicles, and illustrate that a wide variety of vesicles with different sizes and complexities can easily be formed from examining **1** in a simple buffer solution.

### Nonequilibrium Supramolecular Behavior during Oscillations

**Figure 6:**
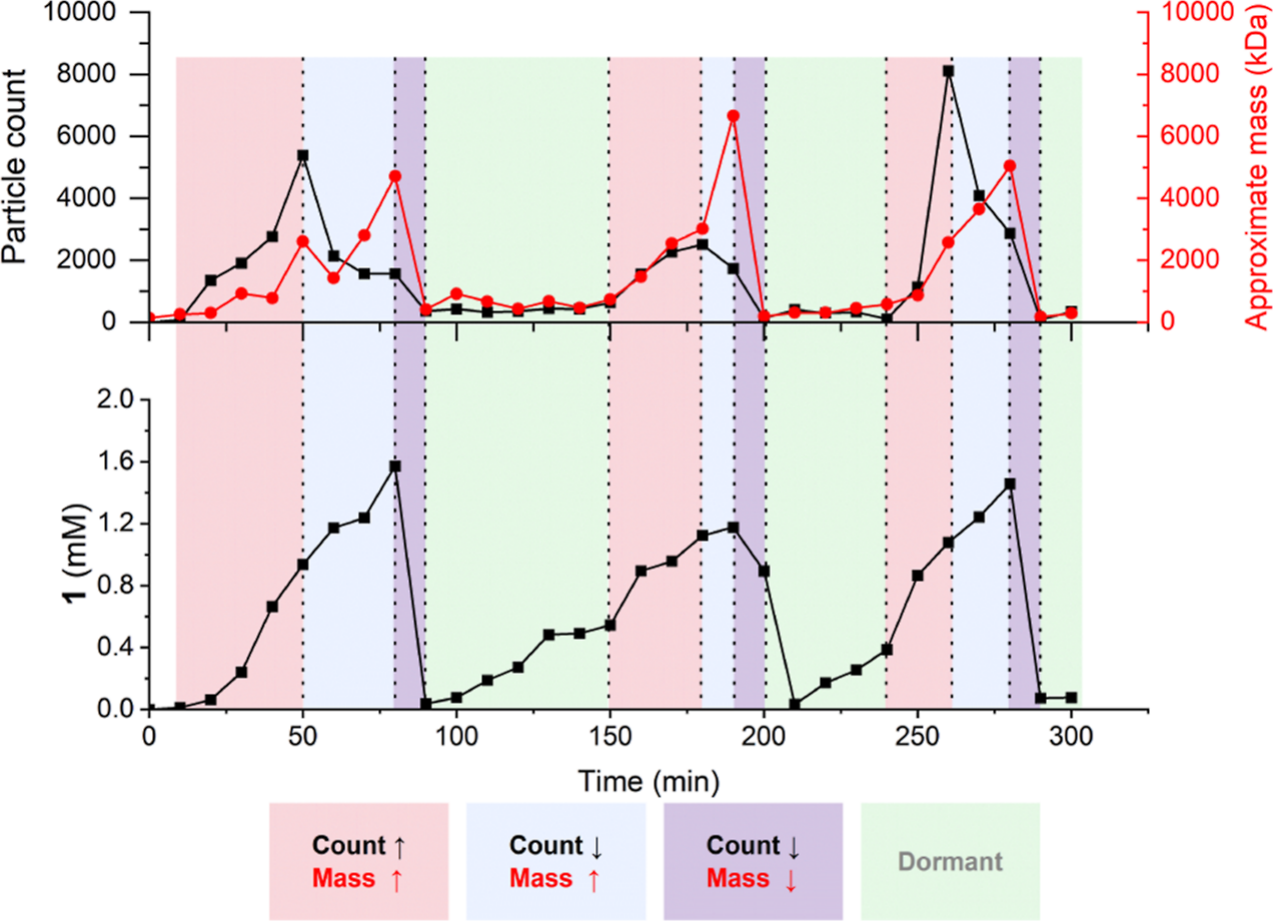
Following the oscillations in **1** where a sample is taken every 10 min and examined by iSCAT and UPLC, four supramolecular stages of the oscillation have been identified and are highlighted by different colors: A “replication phase” (red), a “growth phase” (blue), a “vesicle collapse” phase (purple), and finally a “dormant phase” (green). Lines are drawn to guide the eyes.

Our data shown in Figures 3c,d and 4 show that larger aggregates are favored in later pulses during these oscillation experiments, but this effect is not always observed, and at this stage, it is not clear why this can be inconsistent. It is observed in the data shown in Figure 6, but not in some repeats of that experiment shown in Supporting Information Figure S4.

To further reveal the details of the molecular and supramolecular behavior of **1** during oscillations, a series of reactions were conducted which were monitored by UPLC and iSCAT every 10 min. As seen in Figure 6 (and three repeats of this experiment in Supporting Information Figure S4), the change in the particle count and mass varies throughout the experiment, and the oscillations exhibit four distinct phases.

First, there is a “*replication phase*” (red shaded) where the number and mass of aggregates both increase, suggesting that the vesicles are both simultaneously replicating and growing. Then, the system enters a “*growth phase*” (blue shaded) where the mass of vesicles increases while the particle count begins to drop. During the *growth phase*, the molecular concentration of **1** does not decrease, so the decreased particle count is likely due to the merging of micelles or small vesicles into larger aggregates. Although we cannot be certain, we suspect that replication and growth processes happen simultaneously but dominate the system at different stages and that the “transition” from the “replication phase” to “growth phase” occurs when the number of aggregates in the system makes the probability of collision (and merging) between aggregates more likely, which would cause some vesicles to grow while simultaneously consuming smaller aggregates. It may be that nucleation of supramolecular structures by existing vesicles plays a role in this effect as well, as oligolamellar and multivesicular vesicles^52^ can both be seen from cryo-TEM imaging of **1** in buffer (see Figure 5 and Supporting Information, Figure S9), which would increase the mass of measured particles while potentially offering a mechanism for them decreasing in number without the decomposition of **1**.

The third phase, “*vesicle collapse*” (purple shaded), occurs when **1** decomposes, which (as discussed above) is an autocatalytic process initiated by the depletion of electrophile **3**, and both particle count and mass rapidly decrease in a nonlinear fashion.

Finally, the system then reaches a “*dormant phase*” (shaded green), which starts when the concentration of **1** reaches its minimum and almost no particles are seen. During the dormant phase, molecular **1** increases in concentration until it begins to aggregate and then re-enters the replication phase.

## Conclusions

We have developed a system of oscillating vesicles based on a chemically fueled autocatalytic reaction network. Autonomous oscillations in both monomeric concentrations of amphiphilic disulfide **1** and its aggregates can be observed. The vesicle population undergoes repeating phases of replication, growth, collapse, and dormancy. These dynamic events roughly imitate cell cycles. Rationally designed supramolecular oscillators are rare but could shed light on how biology operates, and our work here shows that consuming chemical energy can drive biologically relevant rhythms and responses that may be relevant to complex dynamic structures at the origins of life and/or early evolutionary processes.

## Supporting Information Available

Oscillation experiments, sampling and UPLC measurements, iSCAT measurements, DLS measurements, synthesis of compounds, UPLC calibration, contrast-to-mass calibration of iSCAT, replications of iSCAT measurements, DLS measurements of **1**, iSCAT of the vesicles of **1** in the steady state, cryo-TEM measurements of vesicles of **1**, and NMR spectra (PDF)
