# Supplementary material for: A Chemical Reaction Network Drives Complex Population Dynamics in Oscillating Self-Reproducing Vesicles
Source: J Am Chem Soc. 2024 Jun 25;146(27):18262–9. doi: 10.1021/jacs.4c00860 (PMC11240260; doi:10.1021/jacs.4c00860)
Supplement: Supplementary file 1 — ja4c00860_si_001.pdf [file ja4c00860_si_001.pdf]

Supporting information

A chemical reaction network drives complex population dynamics in oscillating self-reproducing vesicles

Zhiheng Zhang, Michael G. Howlett, Emma Silvester,

Philipp Kukura* and Stephen P. Fletcher*

Table of contents

Supplementary methods 3
Oscillation experiments 3
Sampling and UPLC measurements 3
iSCAT measurements 4
DLS measurements 4
Synthesis of compounds 5
Supplementary data 8
UPLC Calibration 8
Contrast-to-mass calibration of iSCAT 11
DLS measurements of 1 12
Replications of iSCAT measurements shown in Figure 5 12
iSCAT of the vesicles of 1 in steady state 14
Replication of chemical oscillation with extended time 17
Cryo-TEM measurements of vesicles of 1 17
NMR spectra 19
References 25

Supplementary methods

Oscillation experiments

All biphasic oscillation experiments were conducted in TRIS buffer (0.5 M, pH 9.00). The pH of prepared buffer was adjusted by adding 0.1 M aq. HCl and measured using a Jenway 3510 benchtop pH meter. The final pH was recorded at RT while stirring, to an accuracy of ± 0.01 pH units. For kinetic experiments, an internal standard (3-methyl-2-nitrobenzoic acid, 4 mM) was used to calibrate the concentration measured by UPLC. All aqueous solutions were prepared by ultrapure Milli-Q water and/or HPLC grade organic solvents. 10 mL round bottom flask and oval stirring bars (L= 15 mm, ø= 5 mm) were used in oscillation experiments in order to keep stirring consistent from run to run.

In a typical oscillation experiment, 23 mg compound **4** and 100 mg 1,4-diazabicyclo [2.2.2] octane (DABCO) were dissolved in 4.0 mL 0.5 M TRIS buffer. The solution was sonicated for 20 s to break any undissolved particles. The solution was stirred at 1200 rpm and heated to 50 °C. After the solution was fully heated and in equilibrium, 0.5 mL of compound **2** was added, while 0.3 M H_2O_2 was injected using an AL-1000 syringe pump at 120 µL/h. During the injection, the needle tip was kept within the stirred aqueous layer.

Sampling and UPLC measurements

Samples were taken every 10 or 20 min. by extracting 20 µL of the aqueous layer. The extracted solution was immediately quenched by 0.6 mL maleimide solution (62 mM, 3:1 H_2O: MeCN).

A Waters Acquity ultra performance liquid chromatography (UPLC) was used to analysis the samples. An Acquity UPLC BEH C18 column (130 Å, 1.7 µm, 2.1mm× 50 mm) was used as stationary phase, and the mobile phase was a mixture of water, MeCN and 2% TFA. The proportion is shown in Table 1. The proportion of solvents change in constant rate between different stages. 0.6 mL/min flow speed was used. The concentration of UV active components of the reaction was monitored using a built-in photodiode array (PDA) detector. Peak areas were integrated at a wavelength of 240 nm. Instrument control and data processing were performed using Empower software.

Time	Water	MeCN	2% TFA
0.00-0.05 min	93%	2%	5%
0.06-0.40 min	70%	25%	5%
1.60-2.20 min	0%	95%	5%
2.45-3.00 min	93%	2%	5%

Table 1 UPLC method

iSCAT measurements

The iSCAT experimental set-up is similar to that described by Young et al[1], with a 520 nm diode laser used as the incident light source. Frames were recorded at 1 kHz with an exposure time of 0.98 ms, using a CMOS camera. Focus in the z axis is maintained using an autofocus system relying on the total internal reflection (TIRF) of a 638 nm beam. Instrument control was performed using the custom software written in LabView.

Data processing was performed using the Refeyn Discover MP and Python 3. In brief, differential imaging was achieved by subtracting sets of images temporally offset by a time Δt. The signal-to-noise ratio was then improved by spatially (3 x 3 binning) and temporally averaging the differential images (3 images). Particle detection was performed as described by Young et al[1]. Briefly, diffraction-limited spots were identified by the software, and fitted to the 2D Gaussian function to give the ratiometric contrast value. The possibility of each diffraction limited spot being attributed to more than one particle was excluded by considerations of landing rates similar to those described by Ortega-Arroyo et al.[2] In each 30 s video, the number and contrast of fitted particles corresponding to binding events was counted to quantify reaction kinetics. The mean and median of contrast were calculated by Python 3.

Glass coverslips (24 x 50 mm, VWR) and gaskets were cleaned by sequential sonication in MQ water and 1:1 MQ water: isopropanol (5 min each), and dried under the nitrogen flow. All coverslips and gaskets were prepared on the day of measurement.

A typical oscillation experiment was run and 10 µL of sample was taken every 10 or 20 mins. The samples were diluted by 0.5 mL TRIS buffer immediately and filtered through a syringe filter (PTFE, 0.22 µm, VWR). 5 µL of TRIS buffer was loaded in the gasket before the measurement. 15 µL of diluted sample was loaded in the gasket and mixed with the pre-loaded TRIS buffer and 30 s iSCAT videos were recorded immediately.

DLS measurements

The size of aggregates in oscillation reactions was measured by dynamic light scattering (DLS). A typical oscillation experiment was run and 10 µL of sample was taken per 10 minutes. The samples were diluted by 0.5 mL TRIS buffer (0.5 M, pH 9.00) immediately and filtered through a syringe filter

(PTFE, 0.22 µm, VWR). The filtered samples were injected into disposable plastic cuvettes (ZEN0040). All samples were equilibrated for 30s before measurement.

Analyses were performed using a Malvern Zetasizer Nano ZEN5600 recording particle and molecule size. Instrument control and data processing were performed using Zetasizer software. Measurements were made using an equilibrated heating probe at 25 °C, setting the following parameters for 0.5 M TRIS buffer (calculated internally using the software's complex solvent builder): refractive index = 1.339, viscosity = 1.0780 mPa s. The parameters of POPC were used to approximate the surfactant **1**. Three repetitions of 12 measurements were taken for every sample.

Synthesis of compounds

All commercial chemicals were purchased from Sigma-Aldrich and were used without further purification. Reactions were followed using thin layer chromatography (TLC) on silica gel-coated plates (Merck 60 F254). Detection was performed with UV-light (254 nm), and/or by charring at ~150 °C after dipping into a solution of $KMnO_4$ (1 g/100 mL in ethanol). NMR spectra were recorded on a Bruker 400 (400 MHz) spectrometer in $CDCl_3$. Chemical shifts are given in ppm with respect to tetramethylsilane (TMS) as internal standard. Coupling constants are reported as J-values in Hz. Column chromatography was carried out using Acros silica gel (43-60 µm).

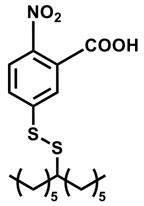

2-nitro-5-(tridecan-7-yldisulfaneyl)benzoic acid (**1**):

5,5'-Dithiobis-(2-nitrobenzoic acid) (332 mg, 0.84 mmol) and trimethylamine (0.36 mL, 0.84 mmol) were dissolved in 4 mL DCM. To this solution was added 7-tridecanethiol (200 mg, 0.92 mmol, 1.1 equiv.). The mixture was stirred for one hour, after which 20 mL of a 1M HCl solution was added. The aqueous layer was extracted with DCM, the organic fractions combined and dried using $MgSO_4$. After removal of the solvent, the crude product was purified using flash chromatography (2% AcOH in DCM), yielding 2-nitro-5-(tridecan-7-yldisulfaneyl)benzoic acid as a light yellow solid (52%)

^{1}H NMR (400 MHz, $CDCl_3$) δ 10.39 (s, 1H, CO_2H), 7.99 (d, J = 2.0 Hz, 1H, $CHCNO_2$), 7.92 (d, J = 8.5 Hz, 1H, SCC*H*CO_2H), 7.82 (dd, J = 8.5, 2.0 Hz, 1H, SCC*H*CH), 2.81 (p, J = 6.5 Hz, 1H, CHS), 1.62 (q, J = 7.5 Hz, 4H, CH_2CHS), 1.50 – 1.36 (m, 4H, alk-CH_2), 1.31 – 1.24 (m, 12H, alk-CH_2), 0.90 (t, J = 7.0 Hz, 6H, CH2CH_3).

^{13}C NMR (101 MHz, $CDCl_3$) δ 170.5 (CO_2H), 147.0 (CNO_2), 145.8 (*C*SSCHCH$_2$), 128.8 (*C*CO$_2$H), 127.1 (*C*CNO$_2$), 126.8 (CSS*C*CO$_2$H), 124.5 (*C*CSSCHCH$_2$), 53.6 (*C*HSS), 33.8 (*C*H$_2$CHSS), 31.6 (alk-*C*H$_2$), 29.1 (alk-*C*H$_2$), 26.7 (alk-*C*H$_2$), 22.6 (alk-*C*H$_2$), 14.0 (CH$_2$*C*H$_3$).

Compound **2** was synthesised according to scheme reported by Engwerda et al.[3] with a few improvements.

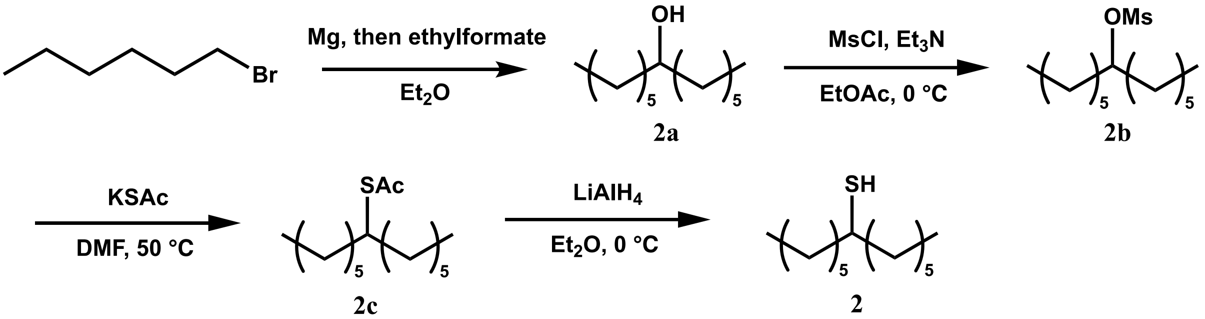

*Scheme S1 Synthesis route for the preparation of **2**.*

<u>7-tridecanol (**2a**):</u>

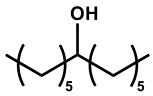

A solution of 1-bromohexane (30.0 mL, 213 mmol) in dry THF (300 mL) in a flame dried flask was cooled to –10 ºC (salt-ice bath), after which magnesium turnings (5.6 g, 230 mmol) were added portionwise (over about 3 min). A solution of ethyl formate (7.05 mL, 87 mmol) in THF was added dropwise (over about 3 min.), while maintaining the temperature below 10 ºC. The reaction mixture was then allowed to warm to RT after which it was stirred for an additional 6 hours. The reaction was quenched with sat. aq. NH_4Cl and then acidified using 1 M aq. HCl. The product was extracted using diethyl ether (3 x 50 mL) and the combined organic extracts were washed with brine and dried ($MgSO_4$). After removal of the solvent, the crude product was purified using flash chromatography (7% EtOAc in hexane) which yielded (after solvent removal) 7-tridecanol as colourless crystals (94%). NMR spectra were in agreement with literature[4].

^1H NMR (400 MHz, $CDCl_3$) δ 3.67 – 3.57 (m, 1H, C*H*OH), 1.52 – 1.35 (m, 4H, alk-CH$_2$), 1.35 – 1.17 (m, 16H, alk-CH$_2$), 0.87 (t, J = 7.0 Hz, 6H, CH$_2$CH$_3$).

^{13}C NMR (101 MHz, $CDCl_3$) δ 72.2 (COH), 37.8 (*C*H$_2$COH), 32.0 (alk-CH$_2$), 29.5 (alk-CH$_2$), 25.8 (alk-CH$_2$), 22.8 (alk-CH$_2$), 14.4 (CH2*C*H$_3$).

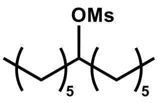

Tridecan-7-yl methanesulfonate (**2b**):

Methanesulfonyl chloride (8.2 mL, 102 mmol) was slowly added to a mixture of triethylamine (26.9 mL, 193 mmol) and 7-tridecanol (19.3 g, 96.4 mmol) in ethyl acetate (250 mL) cooled to 0 ºC. The resulting suspension was stirred for 2 h before dilute HCl (1 M, 100 mL) was added, after which the organic layer was separated, washed with brine and dried using $MgSO_4$. Solvent removal yielded tridecan-7-yl methanesulfonate as a colourless oil (96%).

^{1}H NMR (400 MHz, $CDCl_3$) δ 4.70 (quintet, 1H, CHOMs, J = 6.5 Hz), 2.99 (s, 3H, S(O)2OCH_3) 1.73 – 1.64 (m, 4H, alk-CH_2), 1.46 – 1.23 (m, 16H, alk-CH_2), 0.88 (t, J = 7.0 Hz, 6H, $CH_2C\mathit{H_3}$).

^{13}C NMR (101 MHz, $CDCl_3$) δ 84.5 (COMs), 38.9 ($CHOSO_2\mathit{C}H_3$), 34.6 ($\mathit{C}H_2COH$), 31.8 (alk-CH_2), 29.2 (alk-CH_2), 25.1 (alk-CH_2), 22.7 (alk-CH_2), 14.2 ($CH_2\mathit{C}H_3$).

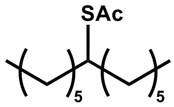

S-(tridecan-7-yl) ethanethioate (**2c**):

Potassium thioacetate (22.5 g, 198 mmol) was added to DMF (60 mL) and then heated to 50 ºC to dissolve all of the solid. The solution was then cooled to room temperature before tridecan-7-yl methanesulfonate (25.7 g, 92 mmol) was added in 3min. The mixture was heated to 50 ºC and vigorously stirred for 5 hours, after which the reaction was allowed to cool to RT. Brine (150 mL) and then diethyl ether (150 mL) were added and the aqueous phase extracted with diethyl ether (3 x 100 mL) and washed with brine (3 x 100 mL). After drying ($MgSO_4$) and removal of the solvent, the crude product was purified using flash chromatography (5% EtOAc in hexane) to yield S-(tridecan-7-yl) ethanethioate as a dark red oil (99%).

^{1}H NMR (400 MHz, $CDCl_3$) δ 3.54 – 3.46 (tt, 1H, CHOAc, J = 5.5 Hz, 7.8 Hz), 2.31 (s, 3H, SC(O)CH_3) 1.64 –1.45 (m, 4H, alk-CH_2), 1.42 – 1.21 (m, 16H, alk-CH_2), 0.88 (t, J = 7.0 Hz, 6H, $CH_2C\mathit{H_3}$).

^{13}C NMR (101 MHz, $CDCl_3$) δ 196.3 (S*C*(O)CH_3) 44.9 (CSAc), 35.0 (*C*H2COH), 31.9 (alk-CH_2), 30.9 (SC(O)$\mathit{C}H_3$) 29.3 (alk-CH_2), 26.9 (alk-CH_2), 22.8 (alk-CH_2), 14.2 ($CH_2\mathit{C}H_3$).

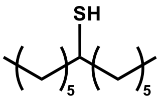

7-tridecanethiol (**2**):

S-(tridecan-7-yl) ethanethioate (6.31 g, 26 mmol), dissolved in dry diethyl ether (20 mL), was added dropwise to a stirred suspension of $LiAlH_4$ (1.98 g, 52 mmol) in dry diethyl ether (100 mL) cooled to 0 ºC (water-ice bath). The mixture was allowed to warm to room temperature at which it was stirred for one additional hour. After cooling to 0 ºC, excess of $LiAlH_4$ was quenched using saturated NH_4Cl, after which the solution was acidified to pH 1 using 3 M HCl. The organic layer was extracted (2x50 mL distilled water), washed with brine (50 mL) and dried using $MgSO_4$. Purification using flash chromatography (100% hexanes, Rf = 0.7) yielded 7-tridecanethiol as a pale tan oil (2.93 g, 52%).

^1H NMR (400 MHz, $CDCl_3$) δ 2.83 – 2.73 (m, 1H, C*H*SH), 1.66 – 1.58 (m, 2H, C*H*CHSH), 1.52 – 1.41 (m, 4H, alk-CH_2), 1.38 – 1.19 (m, 14H, alk-CH_2), 0.89 (t, 6H, CH_2CH_3, J = 7.0 Hz).

^{13}C NMR (101 MHz, $CDCl_3$) δ 41.4 (CSH), 39.2 (*C*H2CSH), 31.9 (alk-CH2), 29.2 (alk-CH2), 27.2 (alk-CH2), 22.8 (alk-CH2), 14.2 (CH2*C*H3).

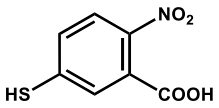

<u>5-Mercapto-2-nitrobenzoic Acid (**4**):</u>

5,5'-Dithiobis-(2-nitrobenzoic acid) (**3**, 1000 mg, 2.53 mmol) was dissolved in 10 mL of an EtOH : H2O (4:1) mixture. The solution was cooled to 0 ºC (ice bath) before $NaBH_4$ (800 mg, 21 mmol) was added carefully and slowly over ~10 min. The mixture was stirred for one hour, before 1M HCl (20 mL) was added and the solution was extracted by degassed CH_2Cl_2 (2 x 30 mL). The organic layer was quickly dried by (Na_2SO_4) and concentrated to dryness to afford bright orange solid (0.80 g, 79%).

^1H NMR (400 MHz, DMSO) δ 14.08 (s, 1H, CO_2H), 8.04 (d, J = 8.6 Hz, 1H, $CHCNO_2$), 7.96 (d, J = 2.2 Hz, 1H, SCC*H*CO_2H), 7.89 (dd, J = 8.6, 2.2 Hz, 1H, SCC*H*CH), 3.36 (s, 1H, SH).

^{13}C NMR (101 MHz, DMSO) δ 165.37 (CO_2H), 146.52 (CNO_2), 141.66 (CSH), 128.97 (*C*CSH), 128.92 (CSH*C*CO_2H), 126.67 (*C*CO_2H), 125.30 (*C*CNO_2).

Supplementary data

UPLC Calibration

The concentrations of the different UV-active species that were analysed using UPLC (see methods section) were calculated based on the calibration curves of Figure S1. All samples were diluted by maleimide solution (62 mM, 3:1 H2O: MeCN) and measured immediately except thiol **2**, which was left overnight to be fully quenched and dissolved.

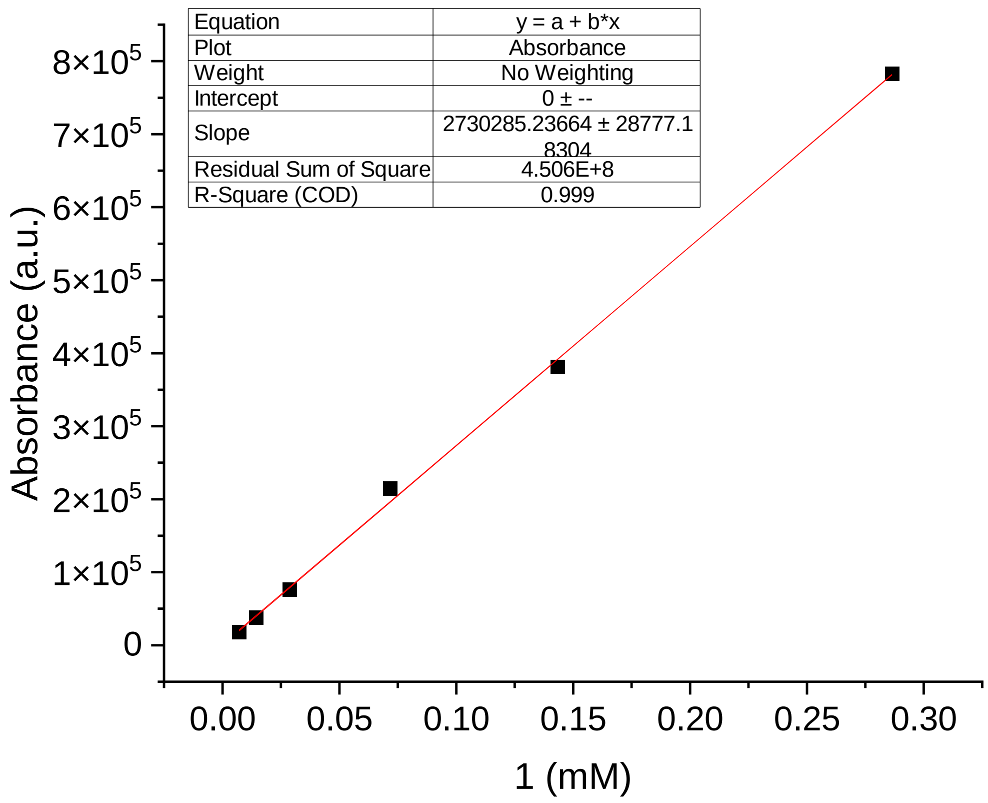
Equation
y = a + b*x
Plot
Absorbance
Weight
No Weighting
Intercept
0 ± --
Slope
2730285.23664 ± 28777.1 8304
Residual Sum of Square
4.506E+8
R-Square (COD)
0.999
Absorbance (a.u.)
0
1×10^5
2×10^5
3×10^5
4×10^5
5×10^5
6×10^5
7×10^5
8×10^5
0.00
0.05
0.10
0.15
0.20
0.25
0.30
1 (mM)

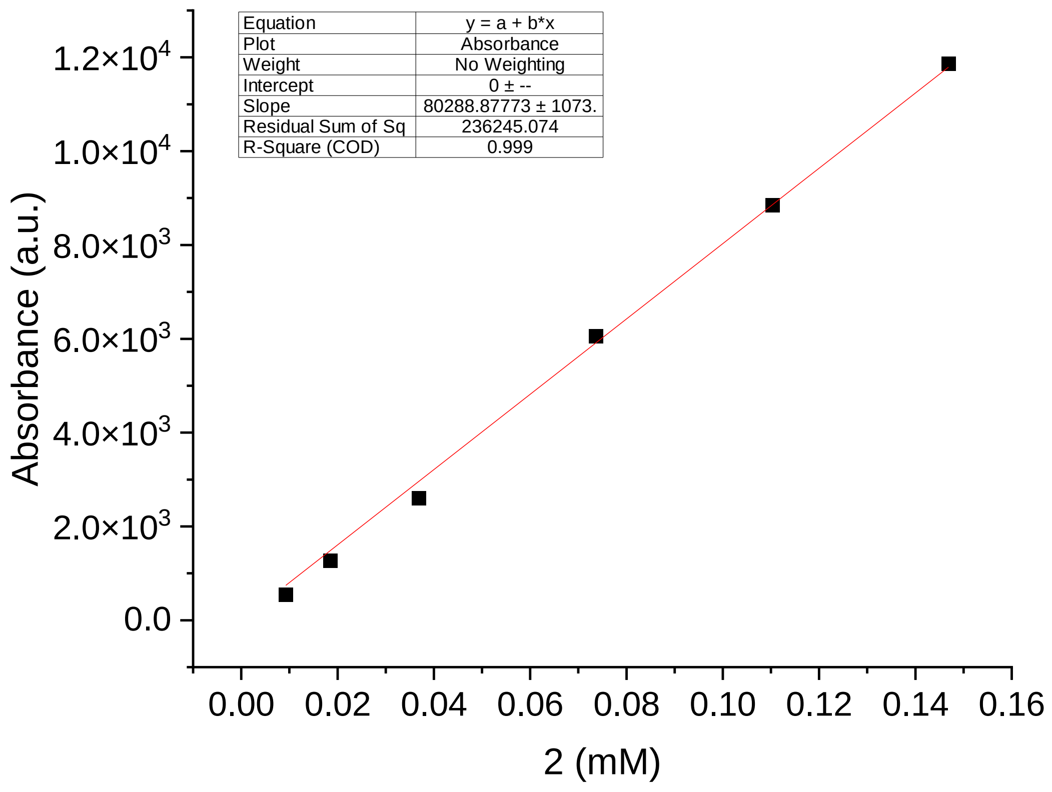
Equation
y = a + b*x
Plot
Absorbance
Weight
No Weighting
Intercept
0 ± --
Slope
80288.87773 ± 1073.
Residual Sum of Sq
236245.074
R-Square (COD)
0.999
Absorbance (a.u.)
0.0
2.0×10^3
4.0×10^3
6.0×10^3
8.0×10^3
1.0×10^4
1.2×10^4
0.00
0.02
0.04
0.06
0.08
0.10
0.12
0.14
0.16
2 (mM)

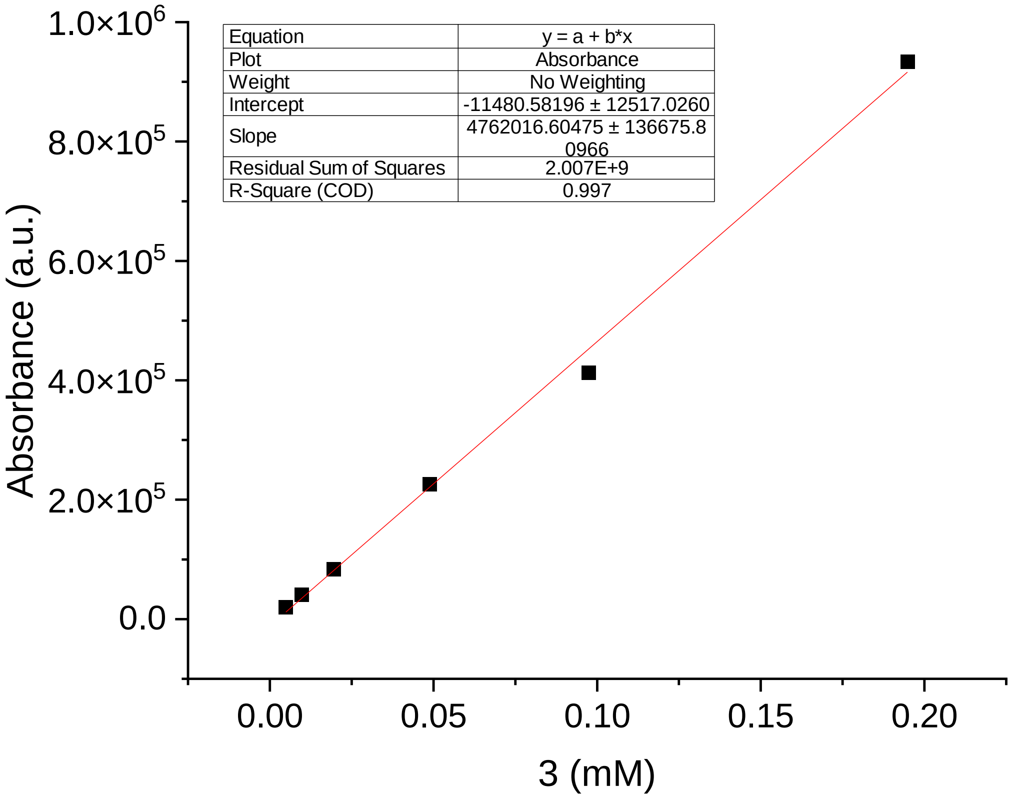
Equation | y = a + b*x
Plot | Absorbance
Weight | No Weighting
Intercept | -11480.58196 ± 12517.0260
Slope | 4762016.60475 ± 136675.8 0966
Residual Sum of Squares | 2.007E+9
R-Square (COD) | 0.997
Absorbance (a.u.)
3 (mM)
0.0
2.0×10^5
4.0×10^5
6.0×10^5
8.0×10^5
1.0×10^6
0.00
0.05
0.10
0.15
0.20

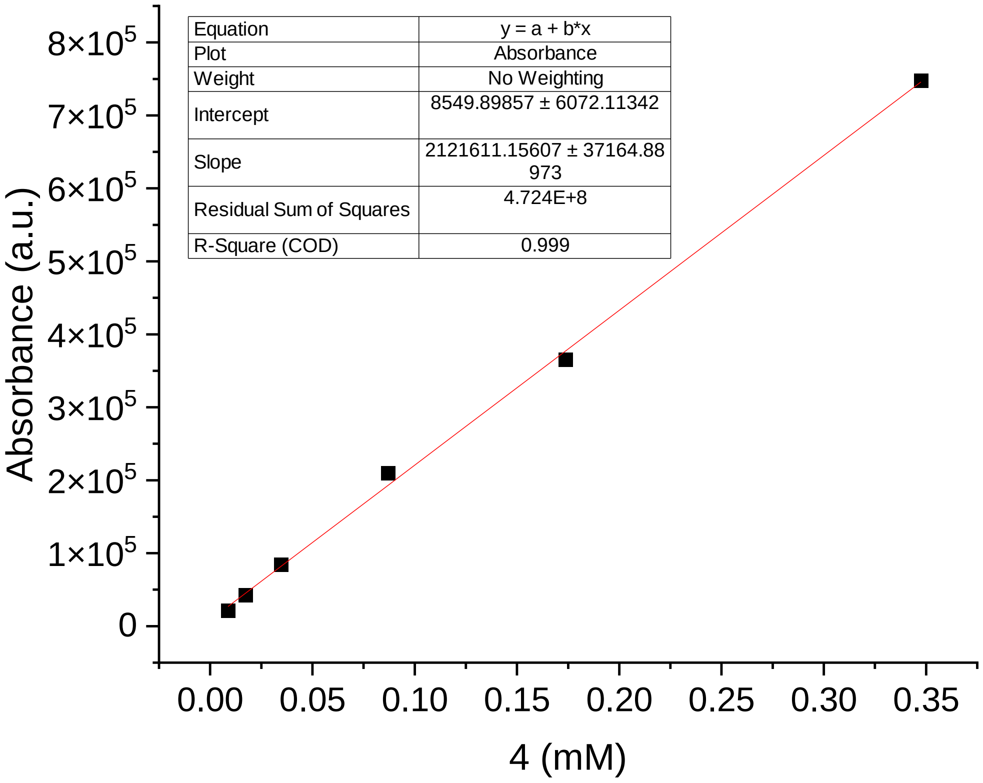
Equation | y = a + b*x
Plot | Absorbance
Weight | No Weighting
Intercept | 8549.89857 ± 6072.11342
Slope | 2121611.15607 ± 37164.88 973
Residual Sum of Squares | 4.724E+8
R-Square (COD) | 0.999
Absorbance (a.u.)
4 (mM)
0
1×10^5
2×10^5
3×10^5
4×10^5
5×10^5
6×10^5
7×10^5
8×10^5
0.00
0.05
0.10
0.15
0.20
0.25
0.30
0.35

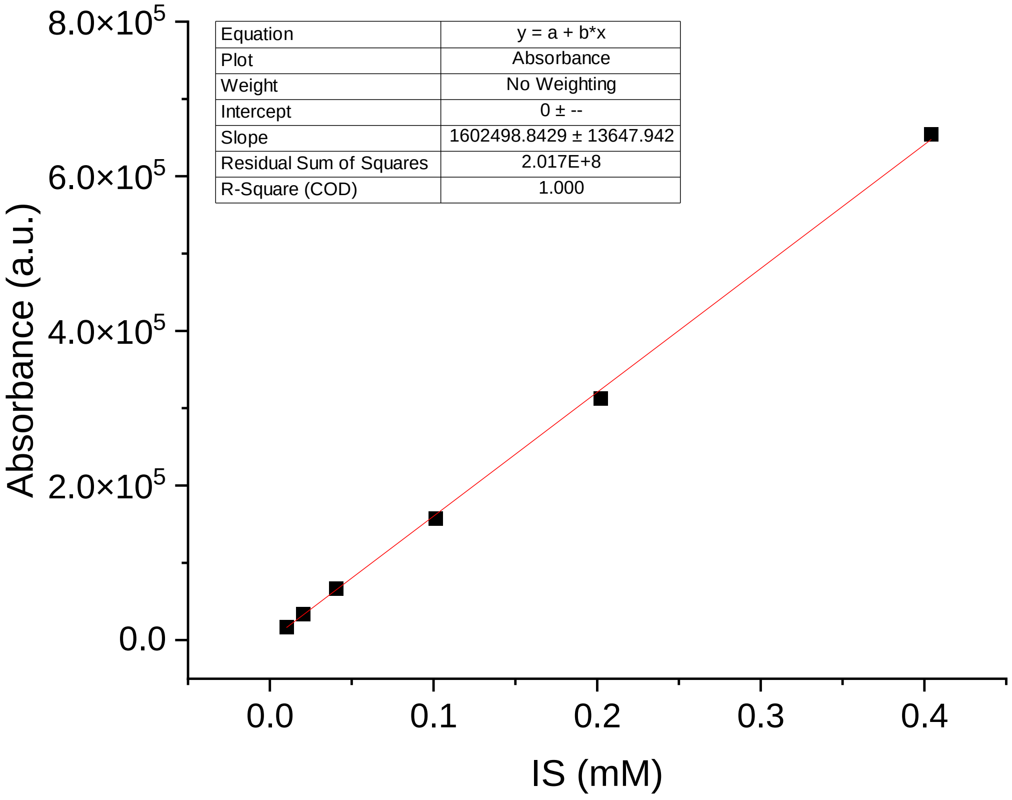

Figure S1 UPLC calibration curves for compounds 1-4 and internal standard.

Contrast-to-mass calibration of iSCAT

Contrast-to-mass (C2M) calibration was performed in commercial PBS buffer solution. The calibration protocol included measurement of a standard protein oligomer solution, with masses of 90, 180, 360, 540 and 720 kDa. Each calibration experiment was analysed using same software as described above. The mean peak contrast was determined in the software using Gaussian fitting. The mean contrast values from the calibration protein solution were then plotted and fitted to a line, y=a + bx, with y-contrast, x-mass, a-intercept and b-C2M calibration factor (Figure S2).

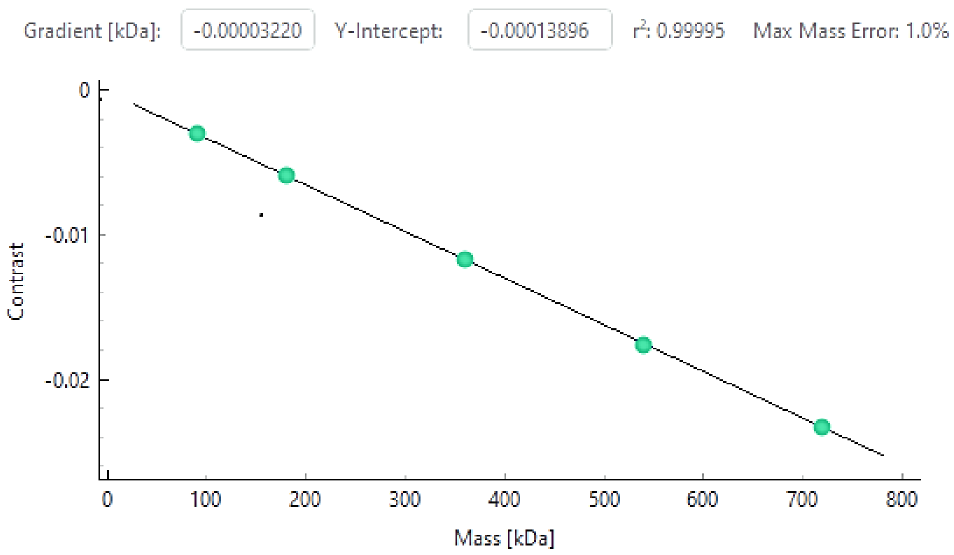

*Figure S2 Calibration curve for the conversion of ratiometric contrast to mass for a known protein oligomer standard, with masses of 90, 180, 360, 540 and 720 kDa. This calibration curve was used to estimate the average mass of the vesicles of **1**.*

DLS measurements of 1

Measurements on **1** in pH9 TRIS buffer gave varying size distribution depending on the concentration. From 2 mM to 0.01 mM, the sizes of vesicles dropped slightly over dilution. The vesicles fully decompose when monomer concentration at < 0.01 mM.

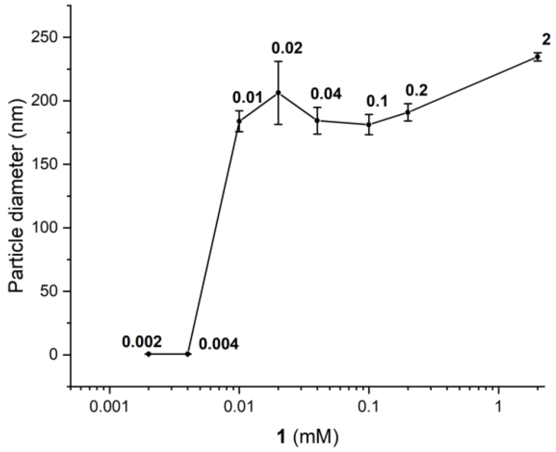

*Figure S3 Number mean diameters for various concentrations of **1** at pH 9.00. Error bars correspond to the standard deviation of the means of three experiments.*

Replications of iSCAT measurements shown in Figure 5

Three repeats of the experiment presented in Figure 5 were conducted. One was done under the same set of measurements, and the other two were allowed to run longer. The four total experiments are consistent with observing four supramolecular stages as shown in Figure 5 in this

oscillating vesicular system. All 4 trials in Figure 5 and here are conducted under as close as possible to identical conditions as described in the oscillation experiments section above.

Experiment A:

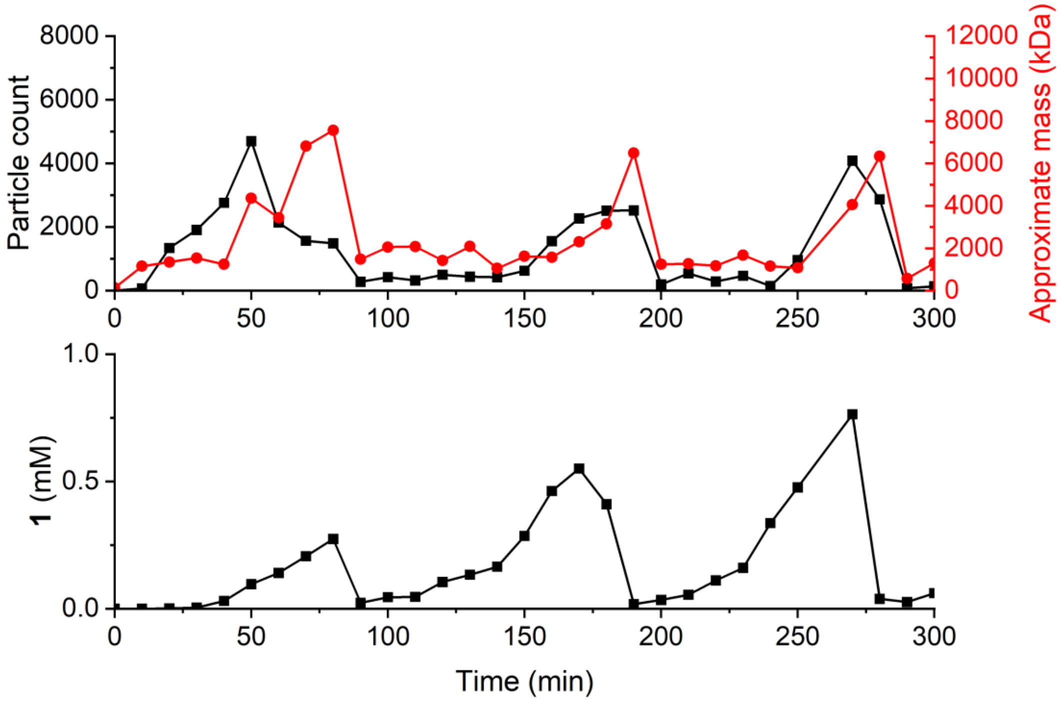

Experiment B:

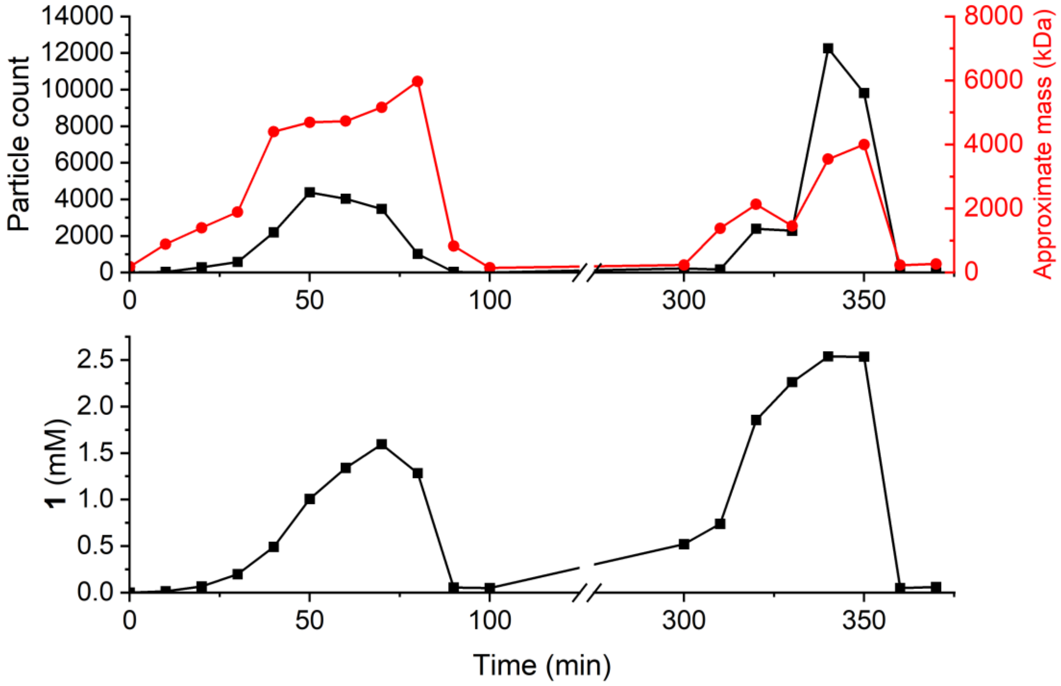

Experiment C:

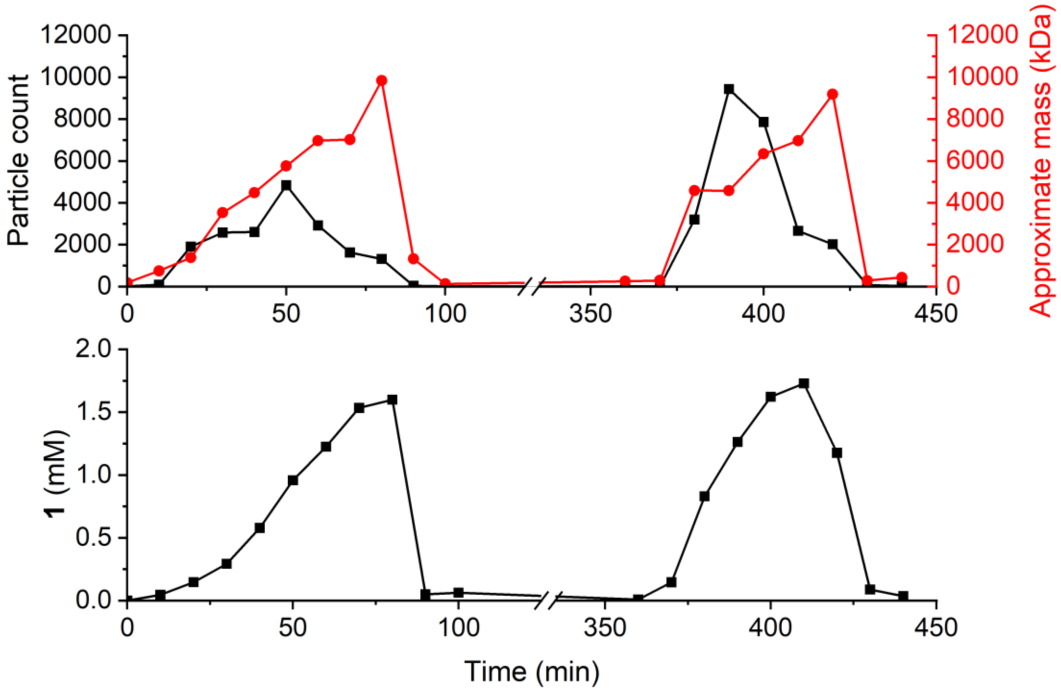

Figure S4. Three repeating experiments of Figure 5.

iSCAT of the vesicles of 1 in steady state

In order to show that the population cycling of vesicles only occurs in oscillation state, the counts and contrast distribution of vesicles of **1** were measured under steady state conditions to make a comparison. The preparation of the vesicles is from the procedure by Engwerda et al.[3] **3** was dissolved in pH 9.0 TRIS buffer to 2.5 mM. Then 2 mL of solution was injected into 10 mL round bottom flask with an oval stirring bar (L= 15 mm, ø= 5 mm). 5.4 mg (5 equiv.) of **2** was added after the solution was heated to 50 °C and stirred to 1200 rpm. The concentration of **1** and **3** was monitored by UPLC (Figure S5). After the concentration of **1** went stable, three 10 µL aliquots were taken at 80, 100 and 130min, diluted by 0.5 mL pH 9.0 TRIS buffer and measured by iSCAT (Figure S6, Figure S7). Figure S6 shows that the count and contrast (approximate mass) of assemblies grew gradually, without showing population cycling.

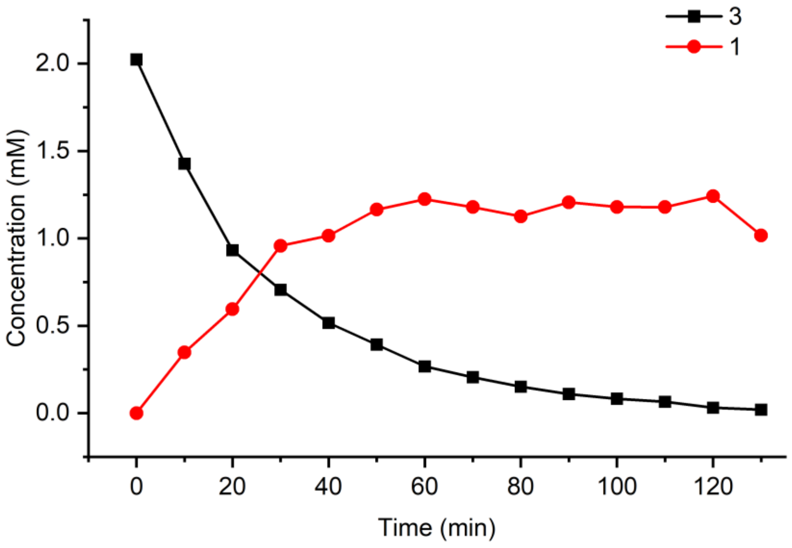

*Figure S5 The concentration changes of **1** and **3** measured by UPLC.*

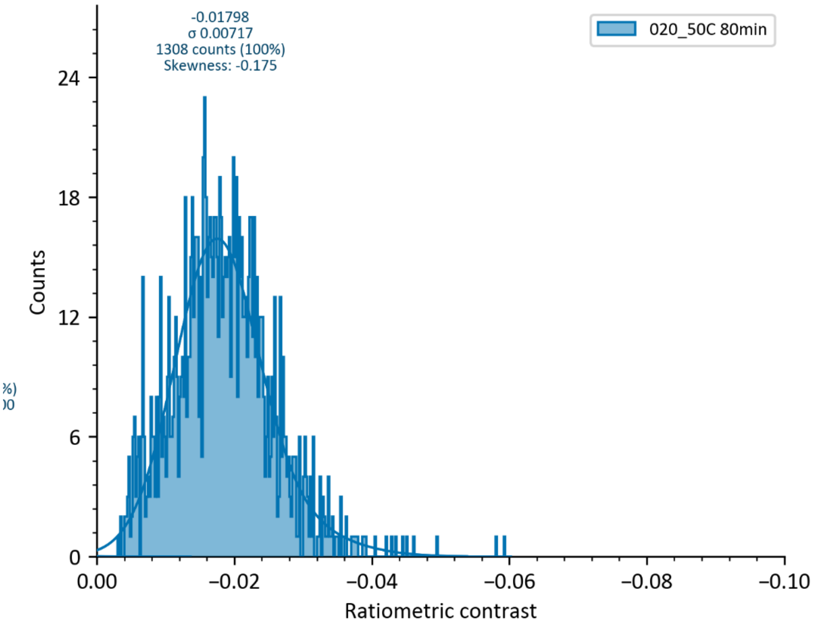

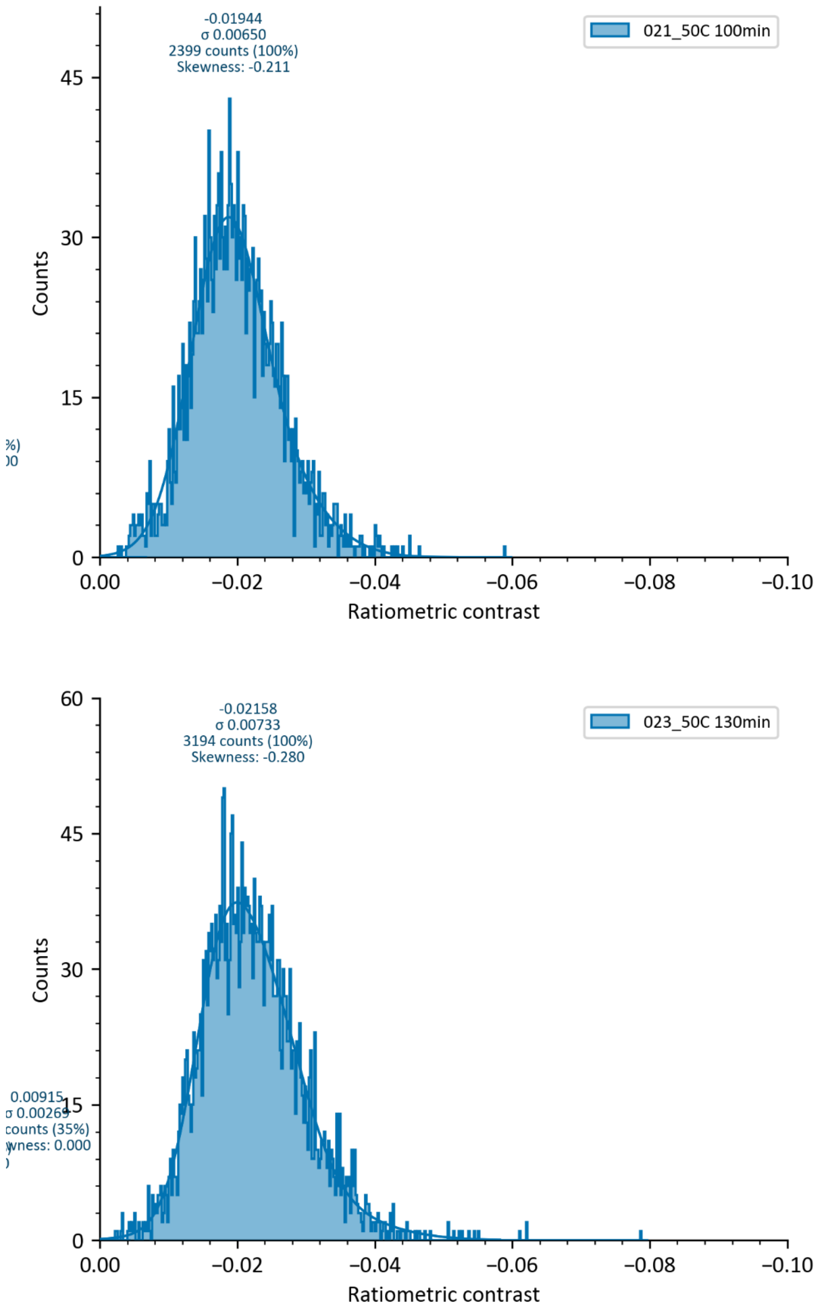

Figure S6 Counts and contrast distributions of vesicles at different times measured by iSCAT

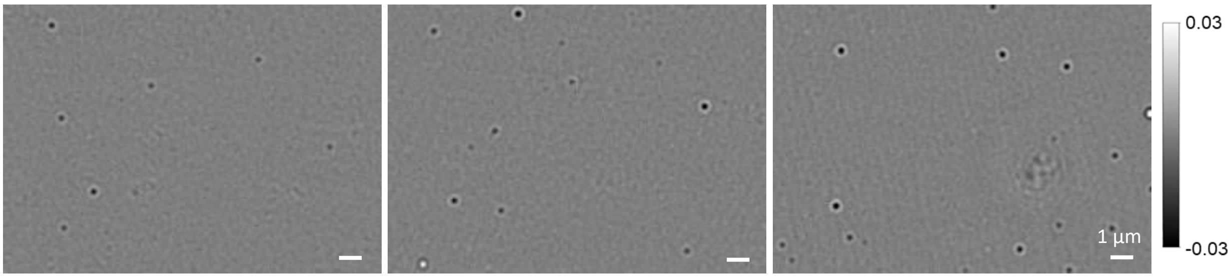

Figure S7 iSCAT images of vesicles at different times, in which contrast scale is set to ± 0.03. The images are taken at 80, 100 and 130 min from left to right.

Replication of chemical oscillation with extended time

The experiment in figure 2b was replicated with longer monitoring time to show reproducibility (Figure S8).

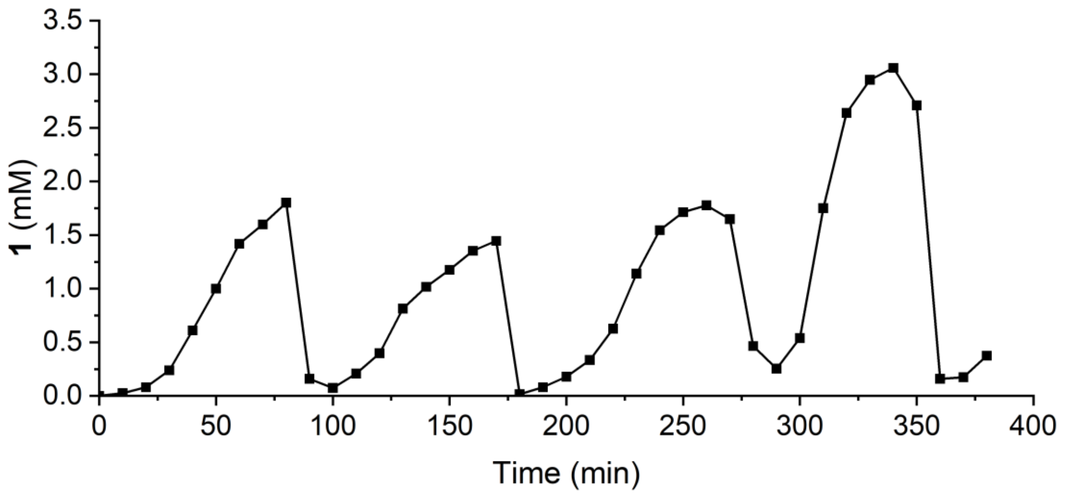

Figure S8 duplicate of figure 2b. Lines are drawn to guide the eye.

Cryo-TEM measurements of vesicles of 1

Plunge Freezing

2.5 mM samples of surfactant **1** were prepared for cryoEM imaging. 4 µL samples were applied to freshly plasma-cleaned Quantifoil 2/1 holey carbon copper-mesh grids (Agar Scientific). Excess sample was blotted away by hand before plunging into a reservoir of propane/ethane with a manual plunger. Grids were stored under liquid nitrogen until imaging.

CryoEM

CryoEM data were recorded on a Titan Krios microscope (FEI, Thermofisher), equipped with a K3 direct electron detector (Gatan) and Quantum energy filter (Gatan). Projection images were collected with SerialEM[5] with a pixel size of 1.1A˚/pixel and the energy-selection slit set to 20 eV. 50 image frames (0.04s exposure/frame) in counting mode were collected at a dose rate of 15 e^-/unbinned pixel/second, giving an overall dose of ~25 e^-/Å^2. A defocus of 4 µm under focus was used to record data. Frames were aligned on the fly in SerialEM. Images were subsequently processed for viewing in FIJI.[6]

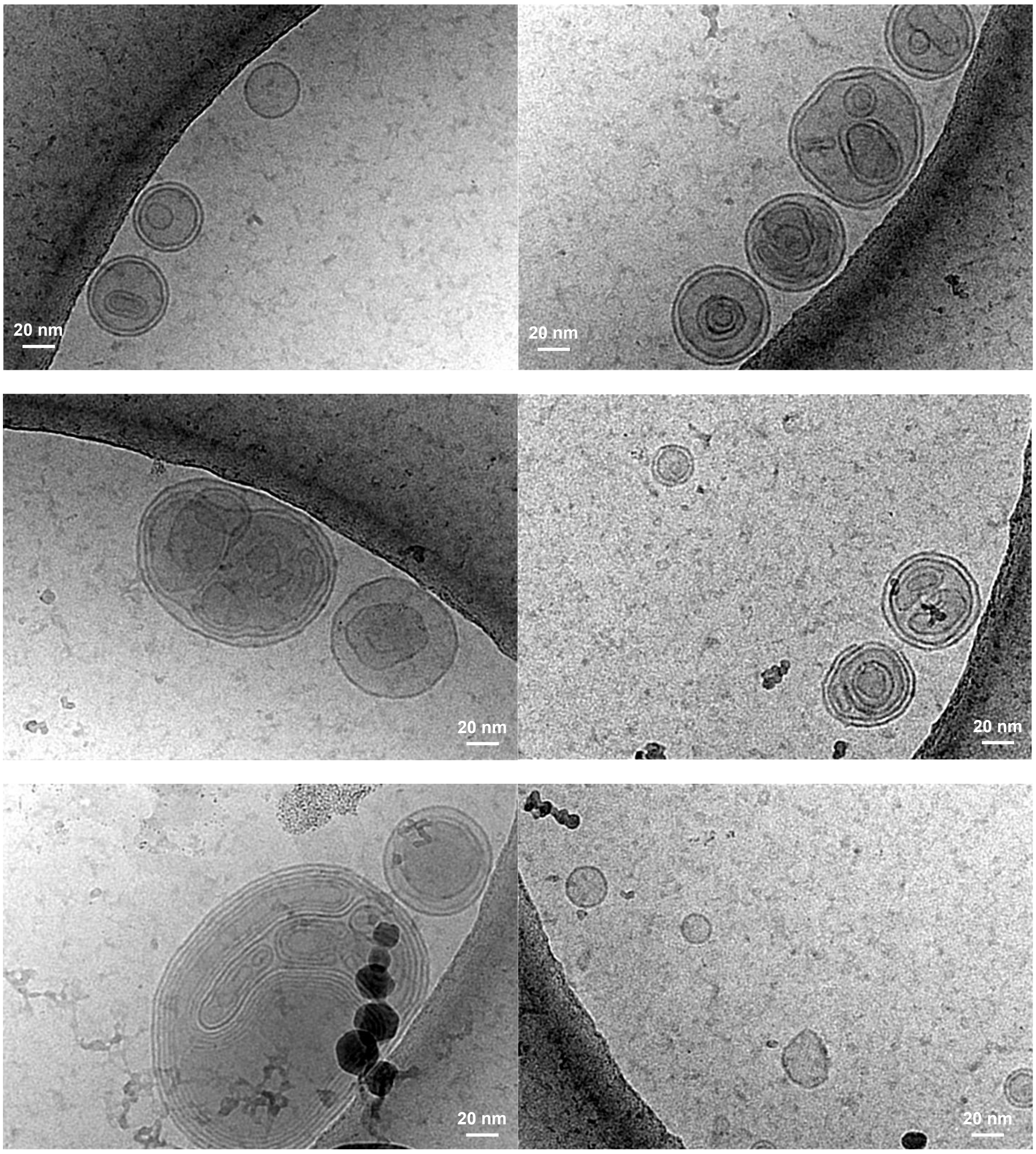

Figure S9. Cryo-TEM images of vesicles composed of surfactants **1**. Images were recorded for 2.5 mM samples of compound **1** in TRIS buffer (0.5M, pH 9.00), showing vesicles of approximately 20 - 190 nm in size.

NMR spectra

2-nitro-5-(tridecan-7-yldisulfaneyl)benzoic acid (**1**):

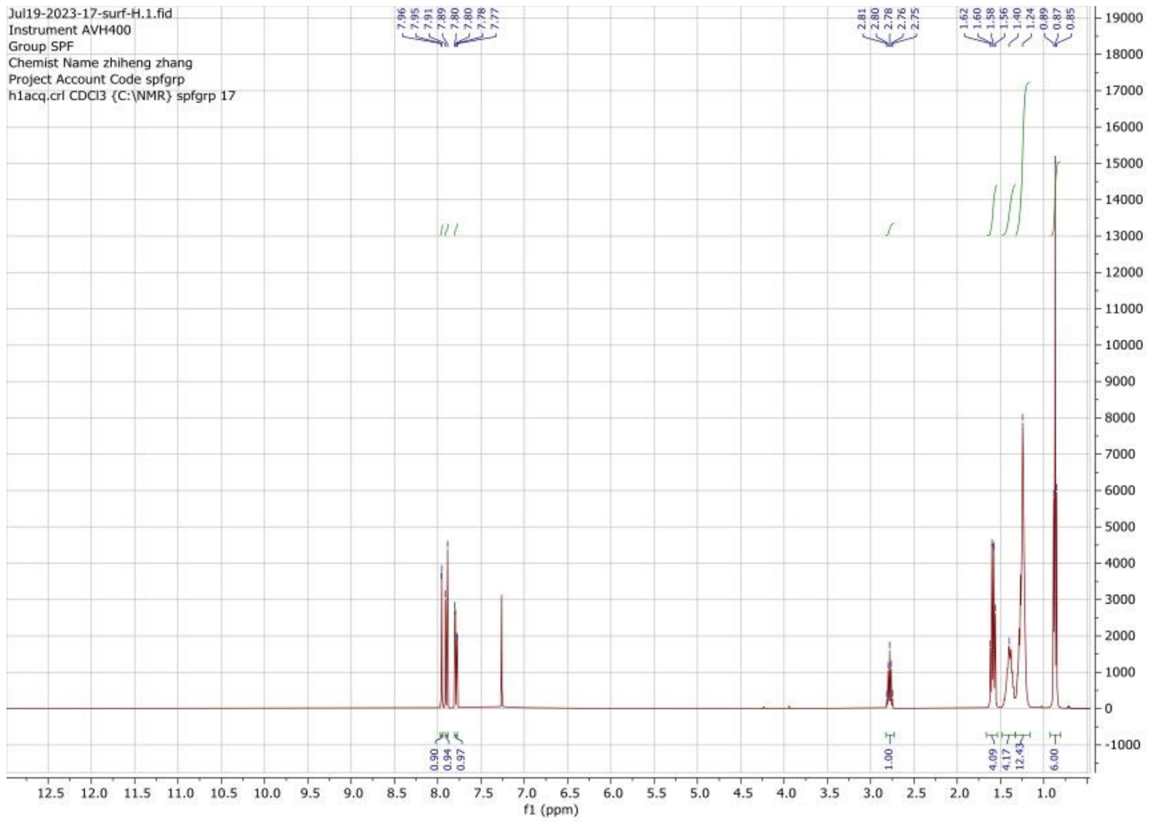

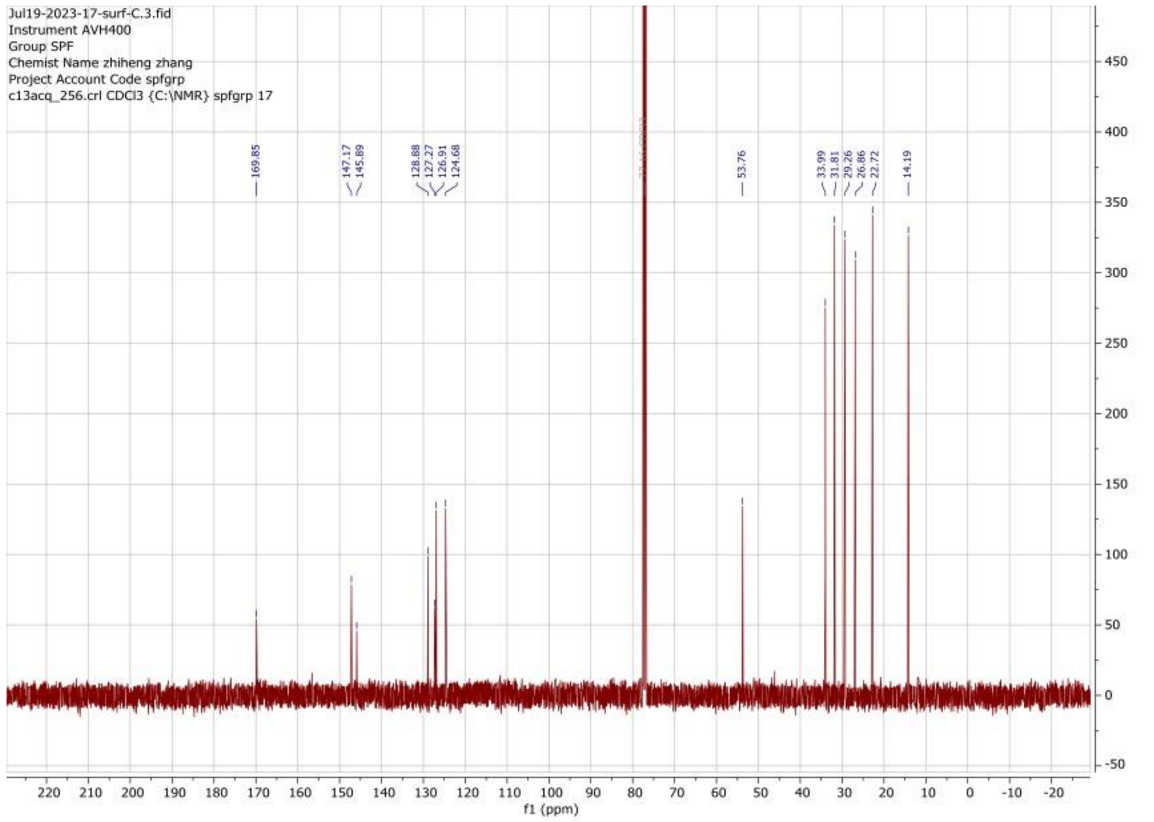

7-tridecanol (**2a**):

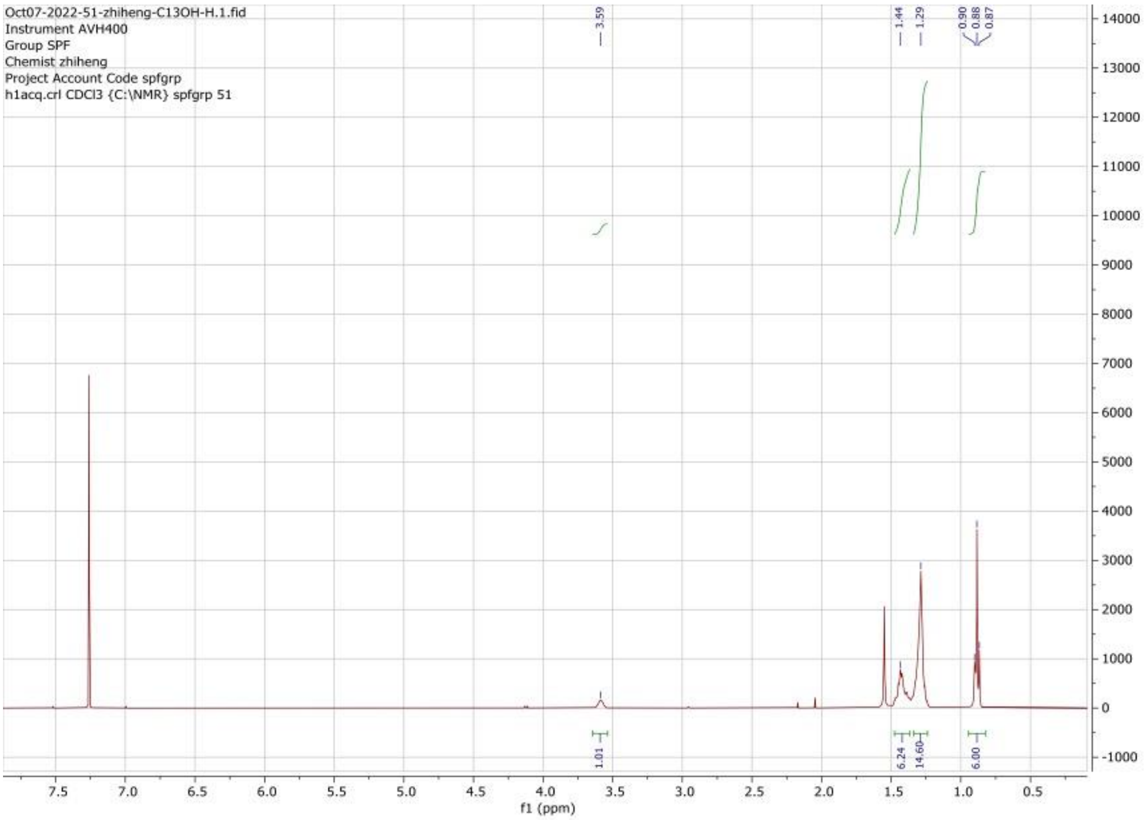

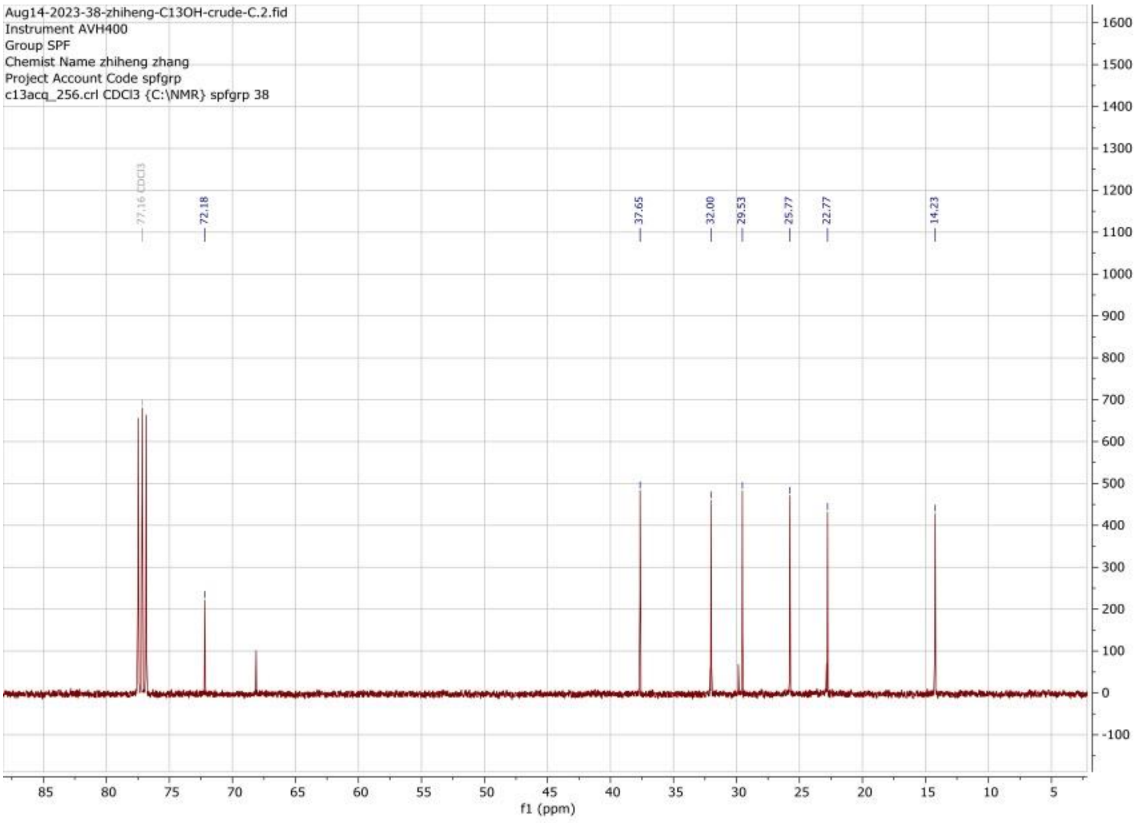

Tridecan-7-yl methanesulfonate (**2b**):

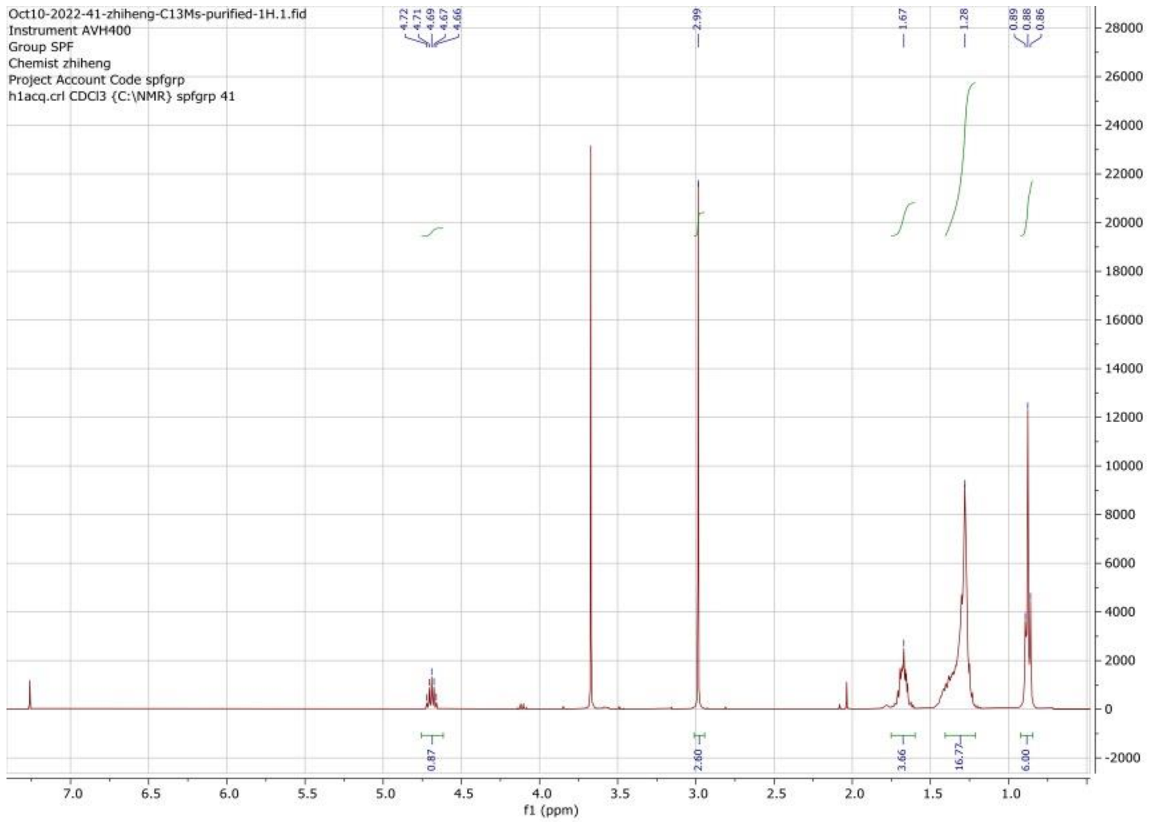

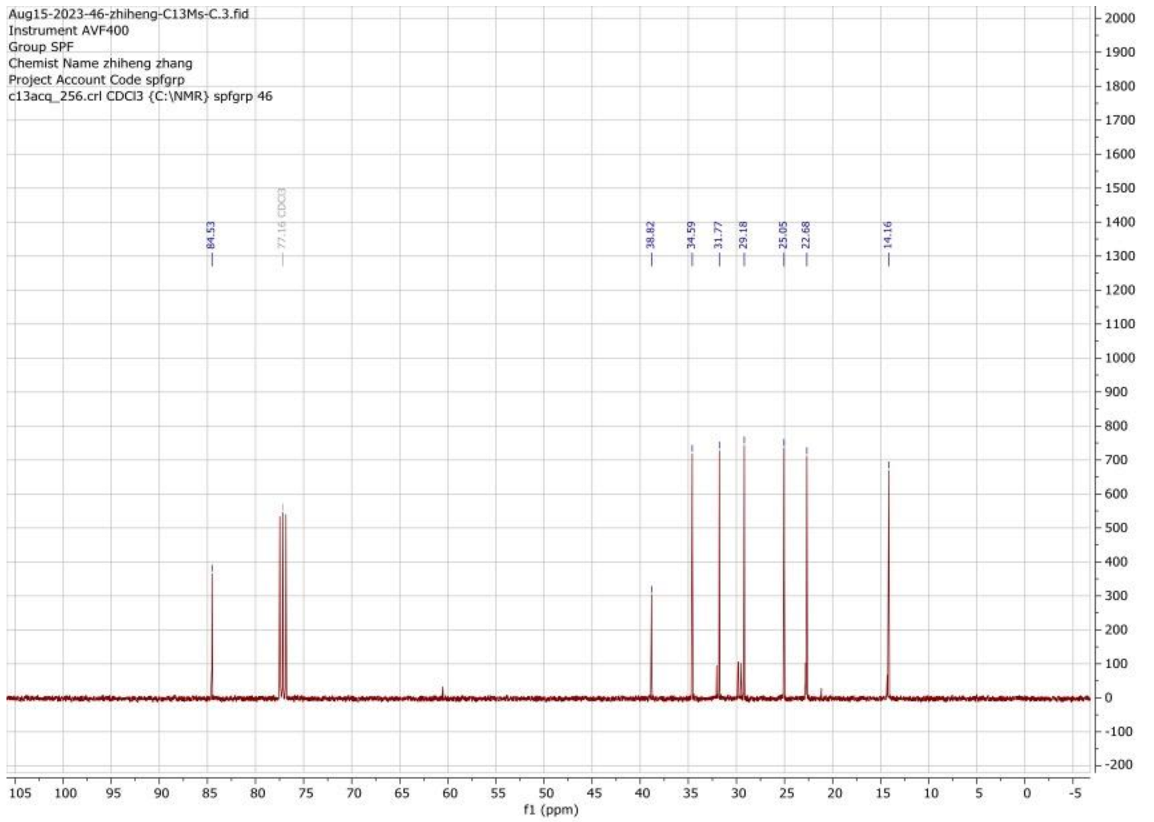

S-(tridecan-7-yl) ethanethioate (**2c**):

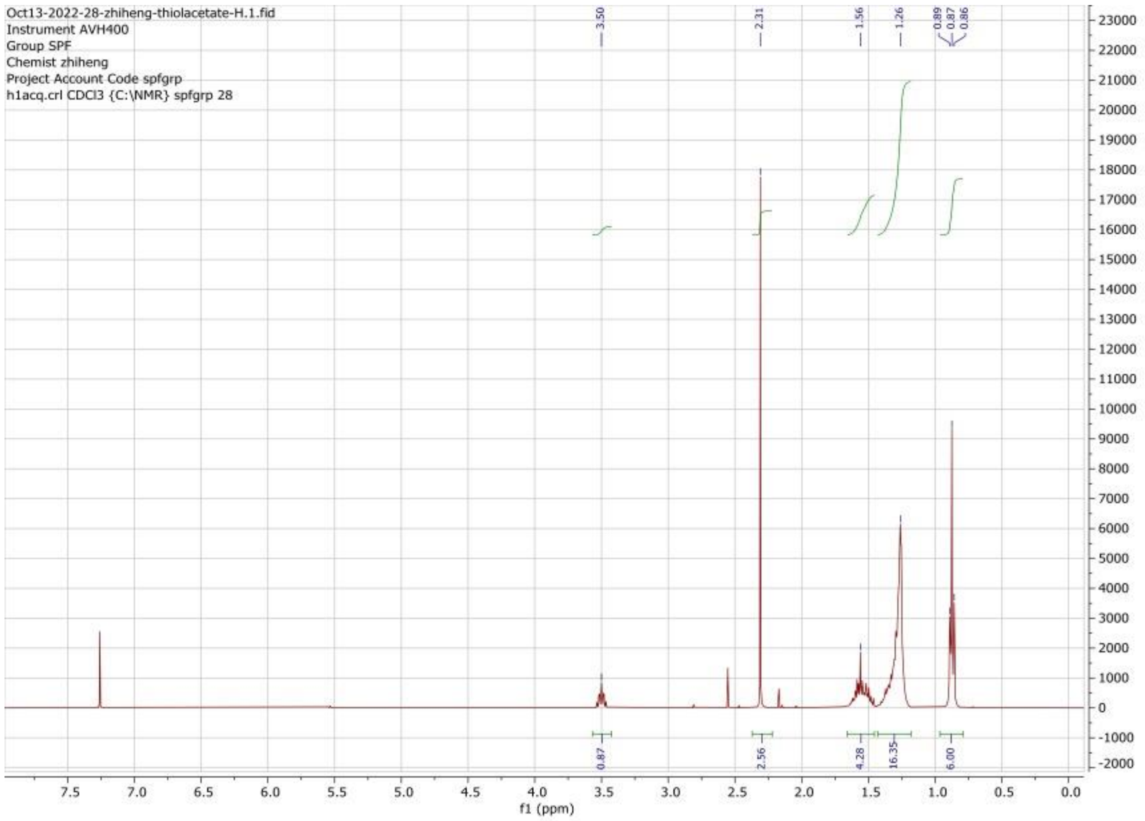

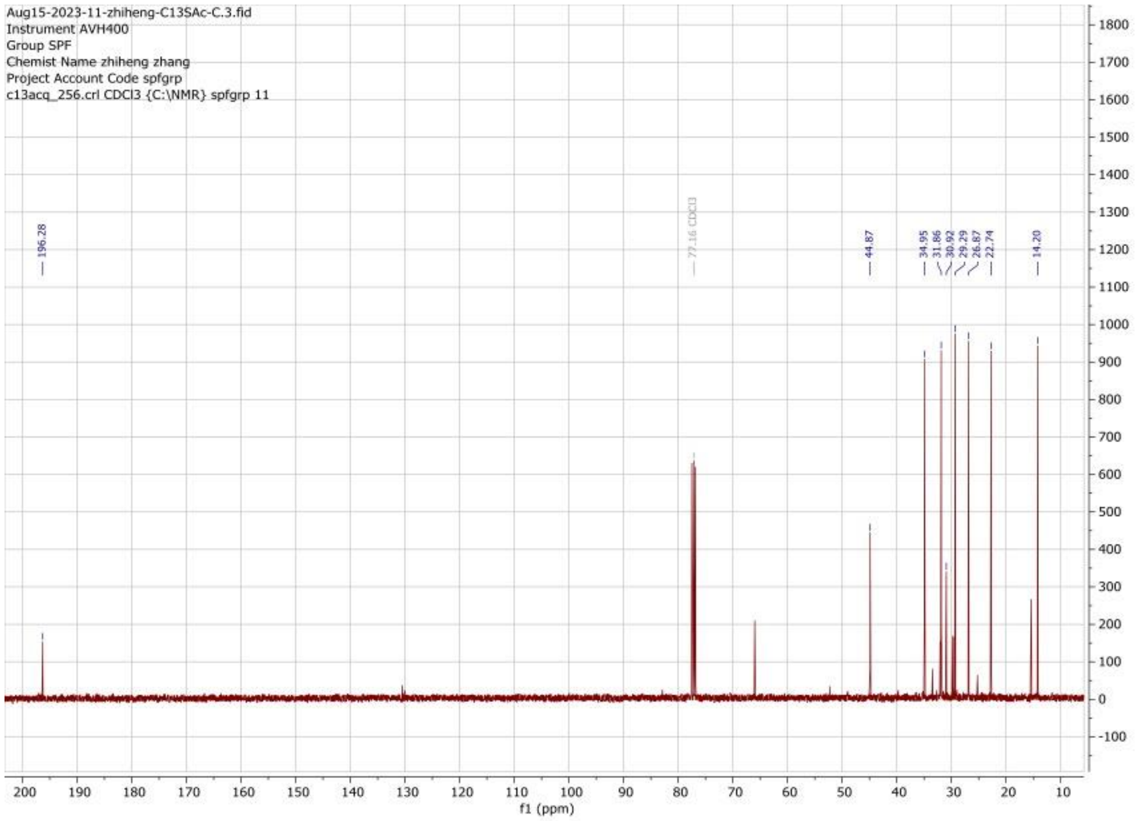

7-tridecanethiol (**2**):

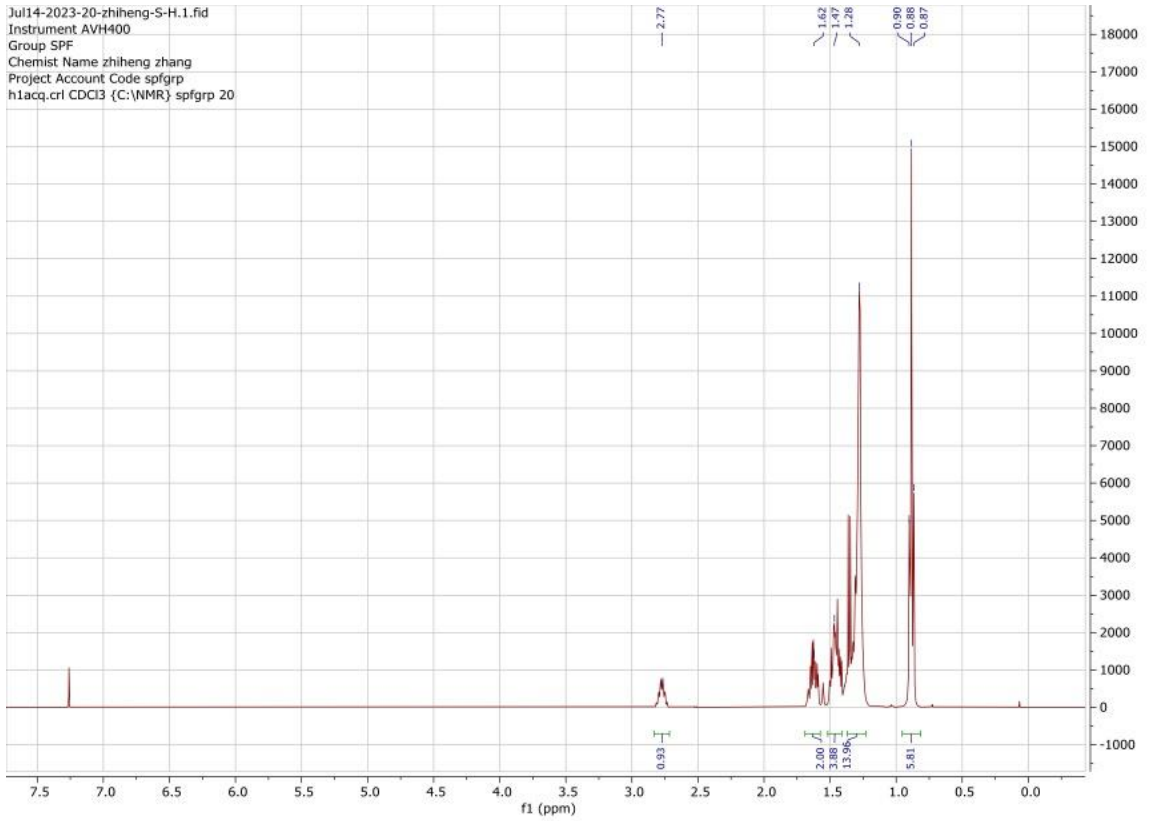

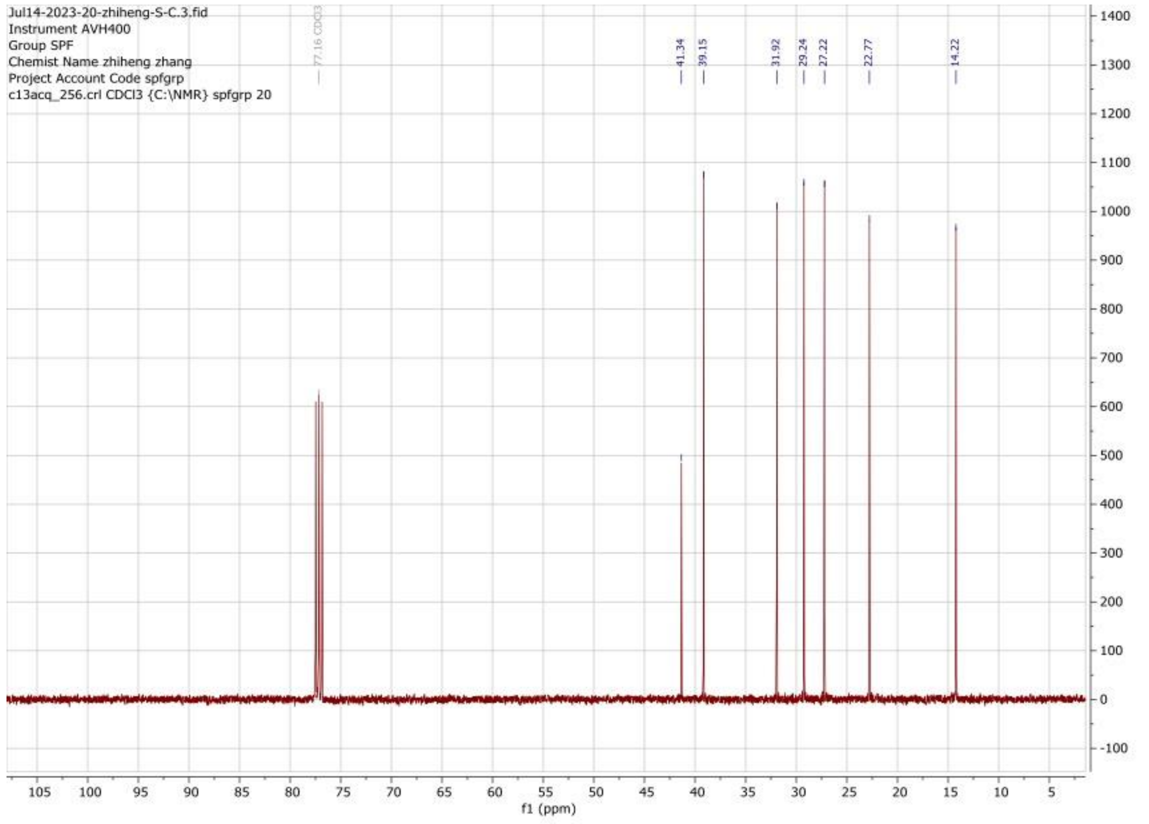

5-Mercapto-2-nitro-benzoic acid (**4**):

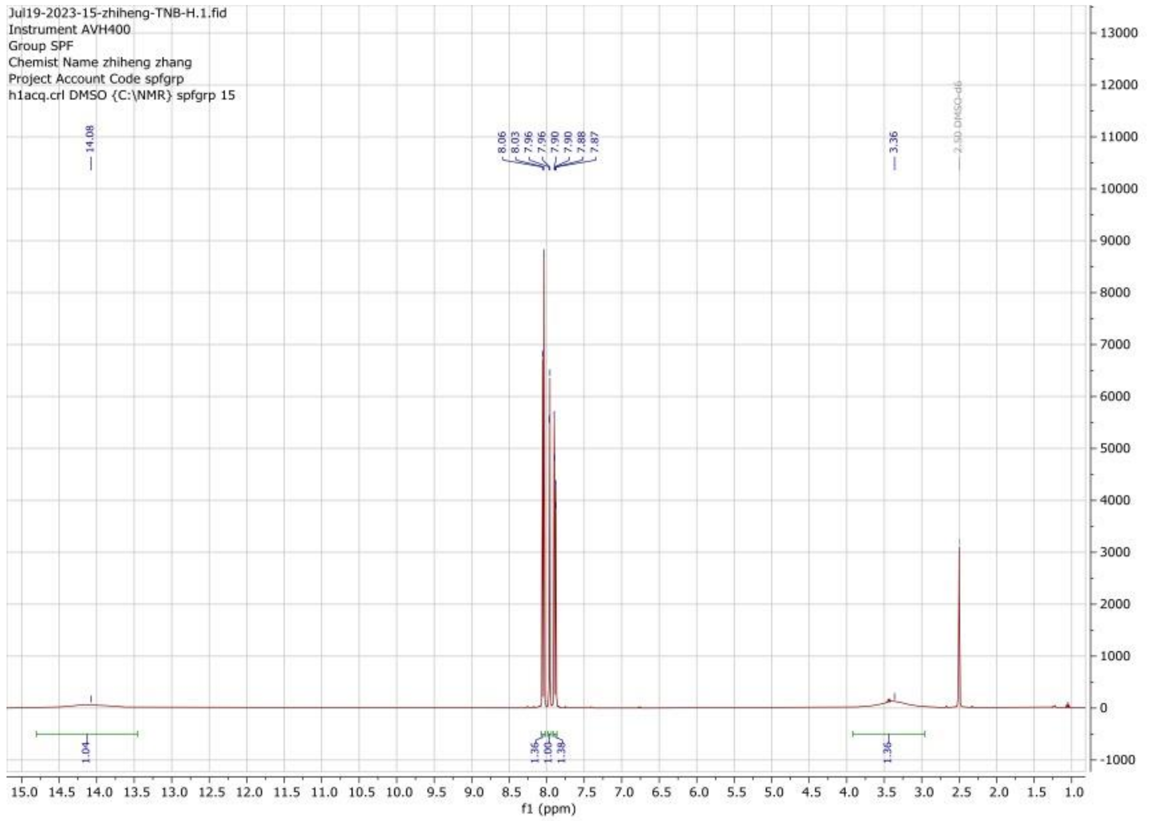

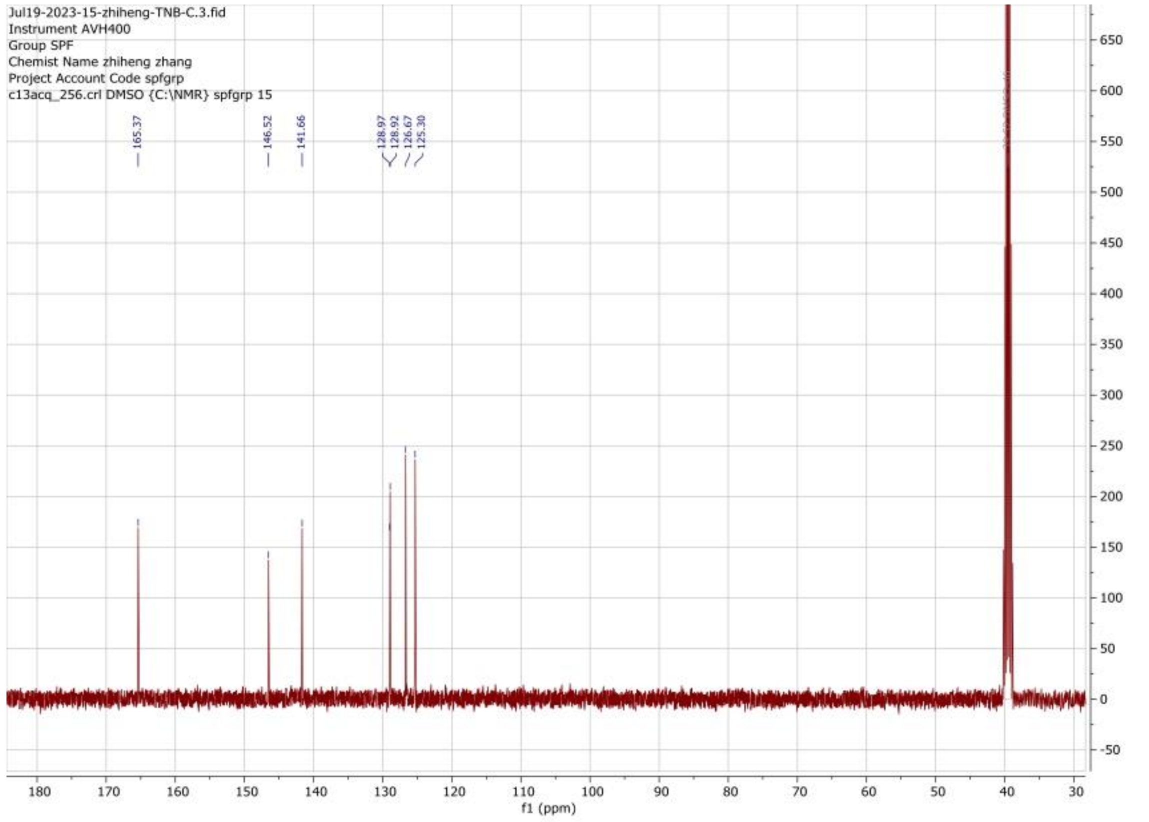

References

(1) Young, G.; Hundt, N.; Cole, D.; Fineberg, A.; Andrecka, J.; Tyler, A.; Olerinyova, A.; Ansari, A.; Marklund, E. G.; Collier, M. P.; et al. Quantitative mass imaging of single biological macromolecules. *Science* **2018**, *360*, 423-427.
(2) Ortega-Arroyo, J.; Bissette, A. J.; Kukura, P.; Fletcher, S. P. Visualization of the spontaneous emergence of a complex, dynamic, and autocatalytic system. *Proc. Natl. Acad. Sci. U. S. A.* **2016**, *113*, 11122-11126.
(3) Engwerda, A. H. J.; Southworth, J.; Lebedeva, M. A.; Scanes, R. J. H.; Kukura, P.; Fletcher, S. P. Coupled Metabolic Cycles Allow Out-of-Equilibrium Autopoietic Vesicle Replication. *Angew. Chem. Int. Ed.* **2020**, *59*, 20361-20366.
(4) Yue, W.; Zhao, Y.; Shao, S. Y.; Tian, H. K.; Xie, Z. Y.; Geng, Y. H.; Wang, F. S. Novel NIR-absorbing conjugated polymers for efficient polymer solar cells: effect of alkyl chain length on device performance. *J. Mater. Chem.* **2009**, *19*, 2199-2206.
(5) Mastronarde, D. N. Automated electron microscope tomography using robust prediction of specimen movements. *J. Struct. Biol.* **2005**, *152*, 36-51.
(6) Schindelin, J.; Arganda-Carreras, I.; Frise, E.; Kaynig, V.; Longair, M.; Pietzsch, T.; Preibisch, S.; Rueden, C.; Saalfeld, S.; Schmid, B.; et al. Fiji: an open-source platform for biological-image analysis. *Nat. Methods* **2012**, *9*, 676-682.
